# Sphingolipid synthesis maintains nuclear membrane integrity and genome stability during cell division

**DOI:** 10.1083/jcb.202407209

**Published:** 2025-07-03

**Authors:** Sunyoung Hwang, William Russo, Jaylah Cormier, Jillian Johnson, Sara Martin, Marica Rosaria Ippolito, Sara Cordone, Rui Li, Lihua Julie Zhu, Stefano Santaguida, Eduardo M. Torres

**Affiliations:** 1 Department of Molecular, Cell and Cancer Biology, University of Massachusetts Chan Medical School, Worcester, MA, USA; 2 Program in Molecular Medicine, University of Massachusetts Chan Medical School, Worcester, MA, USA; 3 Department of Genomics and Computational Biology, University of Massachusetts Chan Medical School, Worcester, MA, USA; 4 Department of Experimental Oncology at IEO, European Institute of Oncology IRCCS, Milan, Italy; 5 Department of Oncology and Hemato-Oncology, University of Milan, Milan, Italy

## Abstract

Lipid synthesis must be precisely regulated to support membrane growth and organelle biogenesis during cell division, yet little is known about how this process is coordinated with other cell cycle events. Here, we show that de novo synthesis of sphingolipids during the S and G2 phases of the cell cycle is essential to increasing nuclear membranes. Indeed, the products of serine palmitoyltransferase (SPT), long-chain bases, localize to the nucleus and are integral components of nuclear membranes in yeast and human cells. Importantly, inhibition of SPT fails to induce cell cycle arrest, causing nuclear membrane collapse and loss of viability in yeast cells. In human cells, this causes abnormal nuclear morphology and genomic instability, evidenced by the increased incidence of nuclear blebs, micronuclei, anaphase bridges, and multipolar mitosis. These results indicate that dysregulated cell division under low sphingolipid availability can drive several disease-associated phenotypes, including aberrant nuclear morphologies and genomic instability.

## Teaser

Lipid synthesis supports membrane growth and organelle biogenesis. Hwang et al. show that synthesis of sphingolipids, mainly long-chain bases, is essential to increasing nuclear membranes during cell division. Failure to make sphingolipids in human cells causes abnormal nuclear morphology and genomic instability.

## Introduction

Maintenance of genome stability relies on proper chromosome segregation during mitosis. In addition to ensuring faithful portioning of genetic content, before cell division, a mother cell needs to increase cellular membranes that will be used by the newly generated daughter cell. Despite advanced knowledge of the mechanisms that regulate the duplication and segregation of chromosomes (Marchal et al., 2019; McAinsh and Kops, 2023) and of the membrane dynamics during nuclear envelope (NE) assembly (Ungricht and Kutay, 2017; Zhao et al., 2023), the regulation of membrane synthesis during cell division remains poorly understood (Storck et al., 2018). Furthermore, the factors determining the relative amounts of different classes of lipid molecules that constitute lipid bilayers in any given cell type are not known (van Meer et al., 2008). A significant hurdle in understanding the regulation of lipid synthesis includes the fact that lipid levels in the cell depend on the generation of lipids via de novo biochemical pathways, the equilibrium of hundreds of bidirectional and interdependent reactions, and the balance between uptake, storage, and utilization of exogenous lipids.

**Figure S1.**
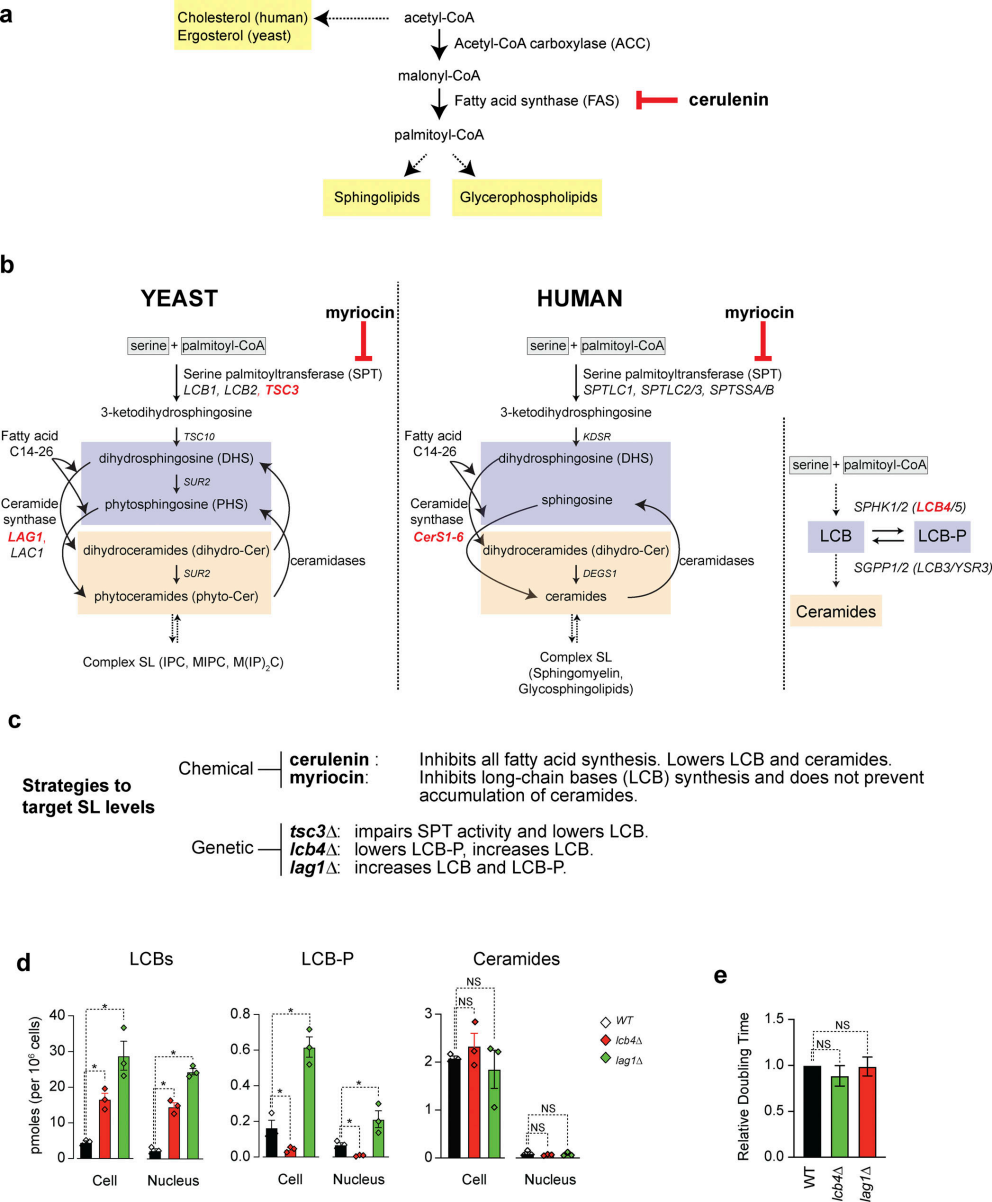
**Synthesis of LCBs determines nuclear shape and volume in yeast. (a)** De novo synthesis pathways of the three major classes of structural lipids in human and yeast cells are conserved. **(b)** De novo synthesis pathway of sphingolipids in yeast and human. DHS and PHS are the main LCB in yeast. DHS and Sph are the main LCB in human. **(c)** Strategies to target sphingolipid levels in yeast cells. **(d)** LCB and ceramide levels in yeast cells. Data are obtained from Hwang et al. (2019). Error bars represent the standard deviations (*n* = 3). *P < 1e-4, one-way ANOVA. NS, P > 0.05. **(e)** Relative doubling time is not affected upon deleting *LCB4* or *LAG1*. Error bars represent standard deviations (*n* = 3). NS, P > 0.05, one-way ANOVA test.

Sphingolipids, glycerophospholipids, and sterols represent three major classes of lipids that make up cellular membranes. In the de novo pathways, acetyl-CoA is used for the biosynthesis of sterols and fatty acids (FA) (Fig. S1 a). Acetyl-CoA carboxylase (ACC) makes malonyl-CoA, which is used by fatty acid synthase (FAS) to make long-chain saturated FA (Fig. S1 a). While the mRNA expression of most enzymes involved in lipid synthesis is not cell cycle–dependent, recent studies point to the upregulation of ACC and FAS during mitosis via posttranscriptional mechanisms (Blank et al., 2017). Nonetheless, it is unclear how changes in enzyme levels or activities lead to specific changes in lipid composition to support cell division or organelle biogenesis.

Sphingolipids are generated by the condensation of palmitoyl-CoA and serine, a reaction carried by serine palmitoyltransferase (SPT), an essential enzyme localized at the nuclear envelope and endoplasmic reticulum (NE/ER) (Fig. S1 b). The product of this reaction, 3-ketodihydrosphingosine, is rapidly reduced to dihydrosphingosine (DHS), which is hydroxylated in yeast to produce phytosphingosine (PHS). A second FA of varying lengths (C_14_–C_26_) is added to DHS or PHS by ceramide synthases to make dihydroceramides (dhCer) or phytoceramides. In humans, dhCer are rapidly desaturated at the ER to yield ceramides (Karsai et al., 2019; Ternes et al., 2002), which can be cleaved by ceramidases to yield sphingosine (Sph). DHS, PHS, and Sph are collectively referred to as long-chain bases (LCBs), and their amine head group is positively charged at physiological pH. Lastly, phytoceramides in yeast and ceramides in humans are converted to complex sphingolipids that differ between these organisms. It is unclear whether the activities of the enzymes in the sphingolipid biosynthesis pathway are cell cycle–regulated. At least in the case of SPT, substrate availability and interaction with ORMDL proteins regulate its activity (Alvarez-Vasquez et al., 2005; Cowart and Hannun, 2007; Schafer et al., 2023; Xie et al., 2023). But whether these modes of regulation are cell cycle–dependent is not known.

In addition to their role as metabolic intermediates, LCBs and ceramides are thought to function as signaling molecules to influence cell proliferation and death (Hannun and Obeid, 2008). Mechanistically, understanding how these essential lipid molecules activate specific signaling pathways is challenging because they are embedded in the membrane, and changing their levels affects membrane properties and elicits a myriad of pleiotropic effects (Hannun and Obeid, 2008). In humans, Sph can be phosphorylated to sphingosine-1-phosphate (S1P), which acts on specific S1P G protein–coupled receptors (S1PR1–5), which control several cellular responses (Ogretmen, 2018).

Recent studies have implicated LCBs in the cellular response to aneuploidy, mainly in cells with an extra copy of an entire chromosome (Hwang et al., 2017; Hwang et al., 2019; Tang et al., 2017; Torres et al., 2010). Lipidome analysis revealed increased levels of LCBs in a series of aneuploid yeast strains relative to euploid controls, with no significant changes observed in complex sphingolipids or glycerophospholipids (Hwang et al., 2017). The levels of LCBs also increase in aneuploid human and mouse cells, as observed in primary fibroblasts with trisomy for either chromosomes 13, 18, or 21 and trisomic mouse embryonic fibroblasts compared with euploid controls (Hwang et al., 2019; Tang et al., 2017). Genetic and biochemical approaches linked altered levels of LCBs with changes in nuclear volume and morphology caused by the presence of an extra chromosome. Indeed, aneuploid cells show aberrant nuclear morphologies. Remarkably, increasing the levels of LCBs suppresses nuclear defects and improves the fitness of aneuploid yeast and human cells (Torres, 2023). These results suggest that LCBs are essential in regulating the nuclear membrane properties in the cell. Here, we analyze the immediate consequences of SPT inhibition in euploid yeast and human cell lines. Our results indicate that the synthesis of LCBs is essential for building the nuclear membrane in yeast and human cells during cell division. Strikingly, failure to synthesize LCBs causes genomic instability in human cells.

## Results

### The levels of LCBs determine the shape and volume of the nucleus in yeast

**Figure 1.**
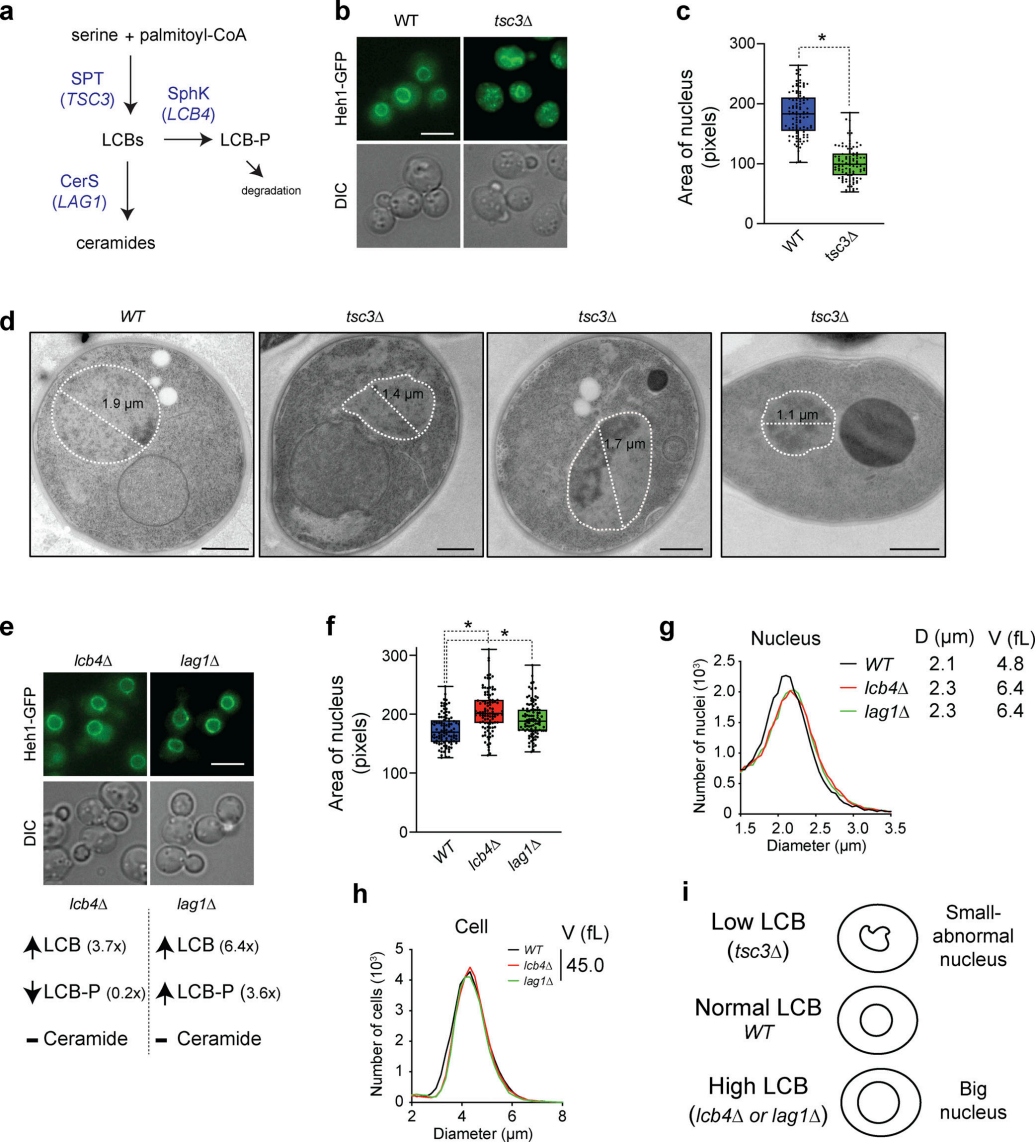
**Levels of LCBs determine nuclear shape and volume in yeast. (a)** De novo synthesis pathway of sphingolipids in yeast. See Fig. S1 b for a more detailed description of the pathway. Genes targeted in our experiments are highlighted in blue. SPT, serine palmitoyltransferase; SphK, sphingosine kinase (*LCB4*); CerS, ceramide synthase (*LAG1*); LCBs, long-chain bases; LCB-P, LCB-1-phosphate. **(b)** Live-cell microscopy of yeast cells expressing Heh1-GFP. DIC, differential interference contrast. Scale bar, 5 µm. **(c)** Area of nuclei of WT yeast and cells harboring the *tsc3∆* (*n* > 100 cells). *P < 1 e-4, unpaired *t* test. **(d)** Representative electron microscopy image of *WT* yeast and *TSC3* deletion (*tsc3∆*). NE contour labeled with a white dotted line for visualization. Scale bars, 400 nm. **(e)** Live-cell microscopy of yeast cells expressing Heh1-GFP harboring the *lcb4∆* or *lag1∆*. Scale bar, 5 µm. Changes in the levels of LCBs relative to WT cells in these strains are shown below (data from Hwang et al. [2019]). **(f)** Area of isolated nuclei of WT yeast and cells harboring the *lcb4∆* or *lag1∆*. Box plots of quantification of nuclear areas (*n* = 200 cells). *P < 1 e-4, one-way ANOVA test. **(g)** Coulter counter profiles of purified yeast nuclei (*n* = 10,000). **(h)** Coulter counter profiles of yeast cells (*n* = 10,000). **(i)** Model of how changing the levels of LCBs affects the nucleus.

To investigate how lowering the levels of LCBs affects the nucleus, we monitored the nuclear morphology of yeast cells harboring a deletion of the SPT regulatory subunit *TSC3* (Fig. 1 a). Deletion of *TSC3* (*tsc3∆*) lowers SPT activity, causing the amounts of LCBs to drop to 30% relative to controls (Hwang et al., 2019). Tagging the endogenous copy of the inner nuclear membrane protein Heh1 with GFP permits visualization of the nuclear membrane in vivo. Live-cell or electron microscopy shows that yeast cells harbor mostly round nuclei and that *tsc3∆* severely affects nuclear morphology (Fig. 1, b and d). Most of the *tsc3∆* cells that can be visualized by live-cell imaging show smaller nuclear volume relative to wild-type (WT) cells in addition to abnormal shapes (Fig. 1 c).

To examine the consequences of increasing the levels of LCBs within the cell, we analyzed nuclear shape and volume in cells harboring two independent mutations that increase the amounts of endogenous LCBs (Fig. S1 c). Of note, adding exogenous DHS or PHS to the growth medium is not a feasible strategy to increase cellular levels of LCBs because they lack specificity, diffuse throughout the cell, and, at micromolar concentrations, hamper proliferation and lower viability. Deletion of the sphingosine kinase *LCB4* (*lcb4∆*) or ceramide synthase *LAG1* (*lag1∆*) causes a four- to sixfold increase in endogenous LCBs (Fig. 1 e and Fig. S1 d). Cell fractionation followed by quantitative lipidomics revealed that the changes in the levels of LCBs accumulate in the nucleus and that the amounts of ceramides are minimally affected (Fig. S1 d). Importantly, these deletions do not affect cell viability or proliferation due to the compensatory activities of paralogs sphingosine kinase *LCB5* and ceramide synthase *LAC1* (Fig. S1 e). Analysis of live-cell microscopy images revealed that nuclei of cells harboring *lcb4∆* or *lag1∆* show a 12–14% increase in surface area relative to WT (Fig. 1 f). The increase in surface area corresponds to a 1.2-fold change in nuclear volume, assuming a perfect sphere. Validating these results, quantifying the volume of purified nuclei using a particle size analyzer revealed that *lcb4∆* or *lag1∆* increased the nuclear diameter to 2.3 µm compared with 2.1 µm in WT cells. These values correspond to nuclear volumes of 4.8 fL in WT and 6.4 fL in *lcb4∆* or *lag1∆* (1.3-fold increase relative to WT, Fig. 1 g). Since *lcb4∆* lowers the phosphorylated form of the LCB (LCB-P) by 80% and *lag1∆* causes a fourfold increase, changes in the LCB-P do not correlate with changes in nuclear volume. In addition, *lcb4∆* or *lag1∆* does not affect cell volume (Fig. 1 h), implicating the synthesis of LCBs in the regulation of the ratio between cell and nuclear volumes. Together, these results show that LCBs are enriched in the nuclear membrane and that lowering their levels causes an abnormal nuclear morphology and smaller volume. Increasing the levels of LCBs leads to increases in nuclear volume without affecting cell volume (Fig. 1 i).

### Inhibition of SPT disrupts nuclear membrane integrity and causes lethality in yeast

**Figure 2.**
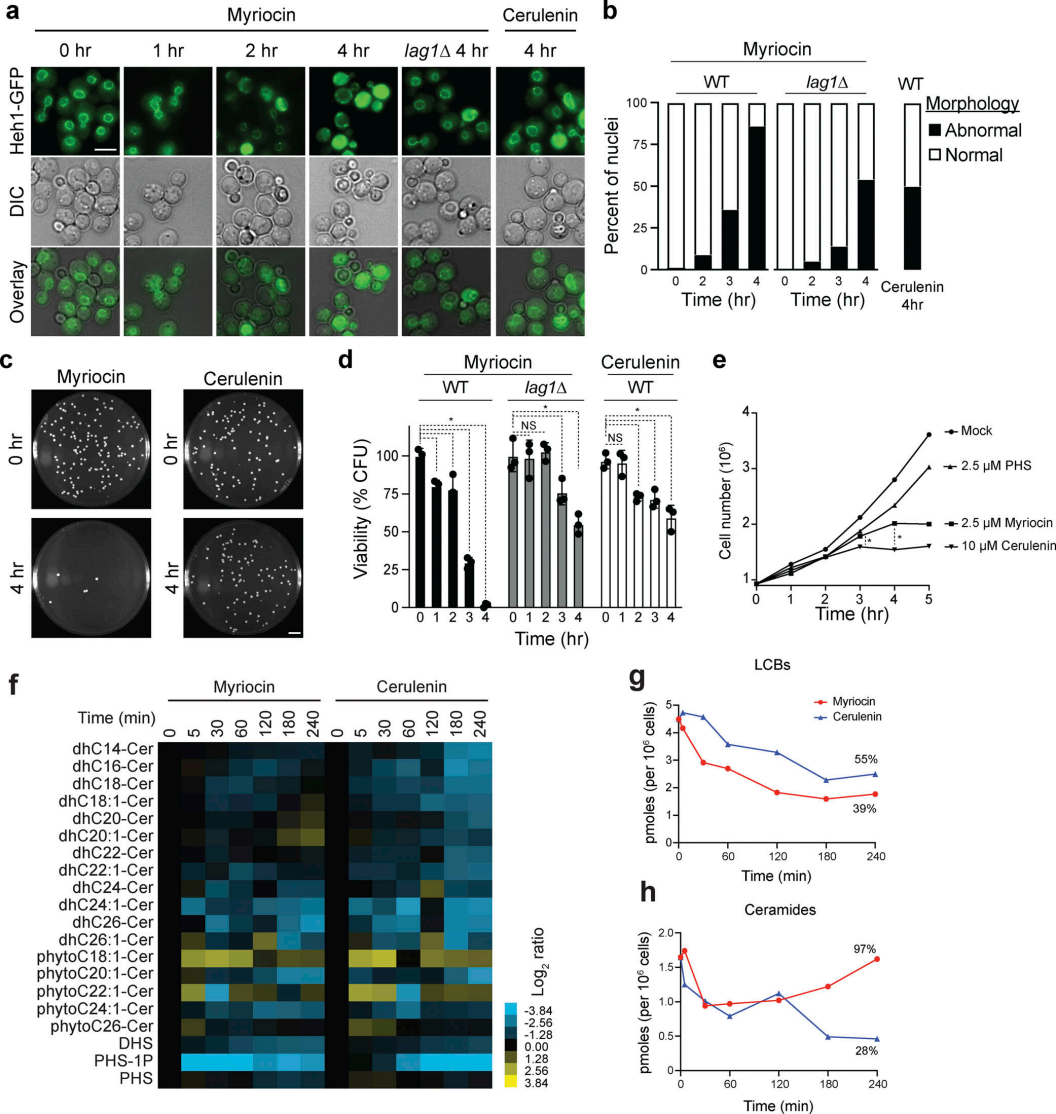
**Inhibition of SPT disrupts nuclear membrane integrity and causes lethality in yeast. (a)** Live-cell microscopy of yeast cells expressing Heh1-GFP treated with 5 µM myriocin or 10 µM cerulenin for the indicated times. DIC, differential interference contrast. Scale bar, 5 µm. **(b)** Quantification of the nuclear phenotype of yeast cells treated with 5 µM myriocin or 10 µM cerulenin (*n* = 200 cells) at indicated times. **(c)** Representative images of CFU assay of yeast cells following treatment with 5 µM myriocin or 10 µM cerulenin at 0 or 4 h. Scale bar, 1 cm. **(d)** Percentage of viable cells quantified by CFU of 200 cells (*n* = 3). Error bars represent the standard deviation. *P < 1 e-4, one-way ANOVA test. NS, P > 0.05. **(e)** Growth curves of yeast cells quantified with a Coulter counter at indicated time points. *P < 1 e-5, paired *t* test. **(f)** HPLC-MS/MS analysis of LCBs and ceramides in yeast cells treated with 5 µM myriocin or 10 µM cerulenin at indicated times. Columns represent experiments, and rows represent lipid species. Log_2_ ratios of the relative lipid levels in comparison with untreated cells are shown. Cer, ceramide; dh, dihydro; DHS, dihydrosphingosine; PHS, phytosphingosine. **(g and h)** Plots of the total lipid classes as a function of time of the lipidome data presented in Fig. 2 f.CFU, colony-forming unit; HPLC-MS/MS, high-performance liquid chromatography–tandem mass spectrometry.

To further characterize the physiological function of LCBs, we monitored the nuclear morphology of yeast cells grown in lipid-free (minimal) medium treated with myriocin, a specific inhibitor of SPT. Cells show abnormal nuclear morphologies within 2 h of myriocin treatment, which is close to the doubling time of yeast in minimal media (Fig. 2, a and b). Within two doubling times, 4 h, nearly 100% of the cells show disrupted nuclear morphologies (Fig. 2, a and b). This phenotype strongly correlates with subsequent loss of viability assessed by a colony-forming unit assay, suggesting that lack of LCBs leads to lethality by disrupting the integrity of the NE in yeast (Fig. 2, c and d). In support of this hypothesis, *lag1∆* cells that accumulate higher levels of LCBs (approximately sixfold) are less sensitive to SPT inhibition (Fig. 2, a, b, and d). While WT cells show complete loss of viability after 4-h treatment with myriocin, *lag1∆* cells show 50% viability and 50% of the cells show normal nuclear morphology (Fig. 2, b and d).

To determine whether general inhibition of lipid synthesis also disrupts the morphology of the nucleus, we targeted FAS with cerulenin. Interestingly, FAS inhibition is better tolerated than SPT inhibition. As expected, cerulenin treatment also causes abnormal nuclear morphologies in yeast, though to a lesser degree than myriocin. At 4 h, 50% of cells treated with cerulenin show normal nuclear morphology (Fig. 2, a and b), and 65% are still viable (Fig. 2, c and d). FAS inhibition lowers the synthesis of sphingolipids and glycerophospholipids as well. Part of the difference between myriocin and cerulenin treatments is that cerulenin inhibits cell division more efficiently than myriocin, indicating that general inhibition of glycerophospholipid biosynthesis causes cells to arrest and stop dividing (Fig. 2 e) (Koberlin et al., 2024). Cell cycle arrest spares the disruption of nuclear integrity, thereby increasing viability.

Exploiting the difference between myriocin and cerulenin, we quantified the effects of these drugs on sphingolipid levels to gain insight into how lipid changes cause lethality and disrupt nuclear morphology. Quantitative lipidomics revealed that the impact of myriocin on lowering the levels of LCBs is more effective than cerulenin (Fig. 2, f and g; and Table S1). At 4 h, myriocin lowers LCBs to 39% levels relative to untreated cells, which causes complete loss of viability, while cerulenin lowers LCBs to 55%, which causes about 30% loss of viability (Fig. 2, d and g). Furthermore, myriocin treatment initially inhibits ceramide synthesis but does not prevent the accumulation of ceramides at later time points, while cerulenin, due to general inhibition of all FA biosynthesis, lowers ceramides to 28% relative to controls (Fig. 2 h). These results indicate that lacking LCBs and not ceramide causes the loss of nuclear membrane integrity and viability. In addition, cells seem to tolerate the reduction of LCBs to a certain degree, suggesting a threshold exists when past nuclear integrity is lost and cells die.

**Figure 3.**
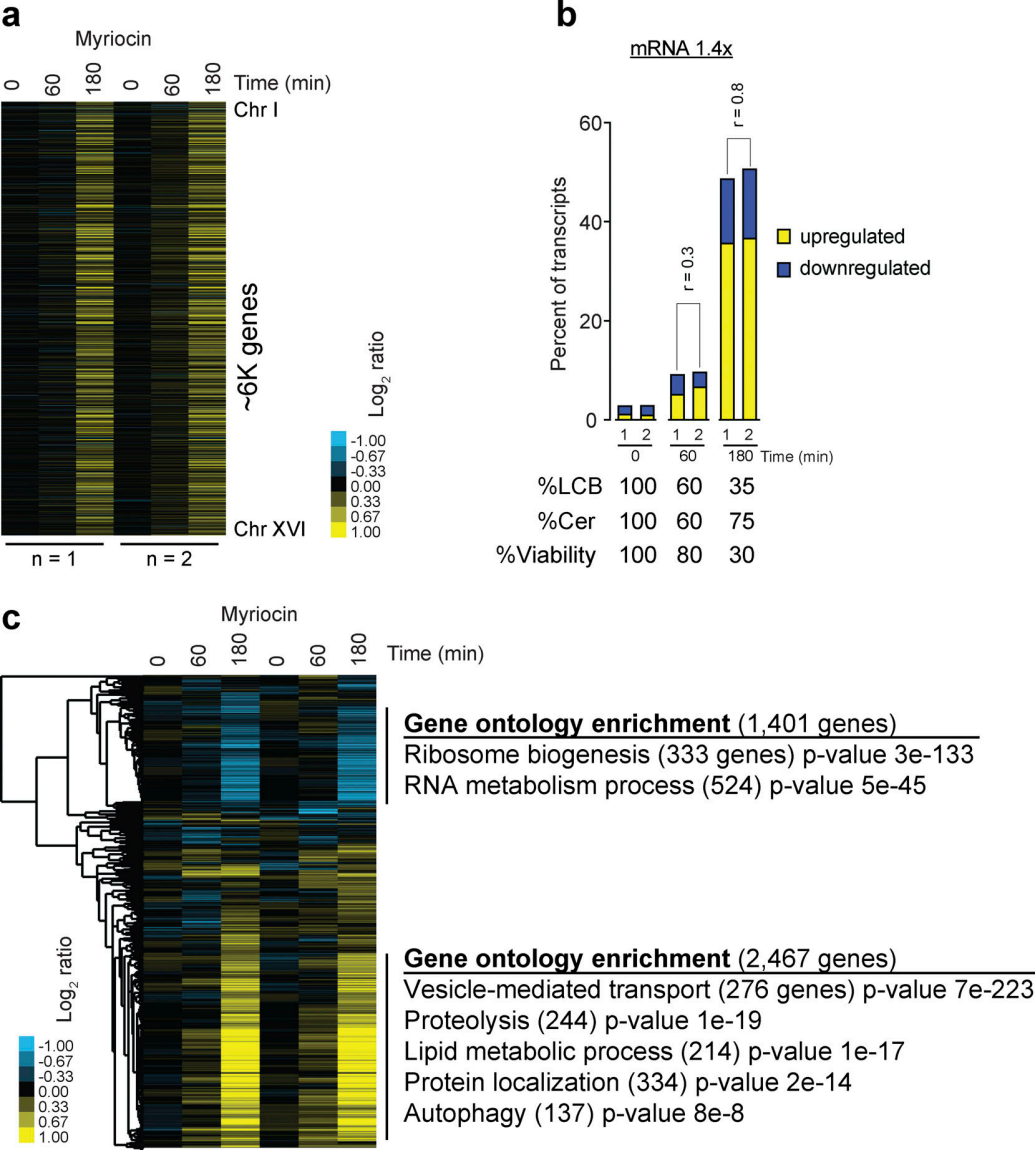
**Gene expression analysis in response to SPT inhibition. (a)** Gene expression analysis of yeast cells treated with 5 µM myriocin for 0, 60, or 180 min grown in batch culture. Experiments (columns), genes (rows), and log_2_ ratios relative to untreated controls (time 0 min) are ordered by chromosome position. Upregulated genes are shown in yellow, downregulated genes are in blue, and genes that do not change are in black. **(b)** Percentage of transcripts that show a 1.4-fold change in levels relative to untreated controls in 0, 60, or 180 min. Relative levels of LCB, ceramides, and viability at each time point are shown below. Pearson’s correlation coefficient r is shown. **(c)** Hierarchical clustering of the gene expression data presented in Fig. 2 b.

To investigate whether cells elicit a specific gene expression program in response to the depletion of LCBs, we performed RNA-seq of cells treated with myriocin for 1 h when viability was minimally affected and the levels of LCBs dropped to 60%. We also performed RNA-seq of cells treated with this drug for 3 h when cells started to lose viability, and the levels of LCBs were at 35% (Fig. 3 a and Table S2). Analysis of the transcriptional changes within 1 h of treatment does not show significant changes in gene expression (Fig. 3 b). Under a stringent cutoff of 1.4-fold change in expression, the list of genes that change is not enriched for a particular cellular process or function according to gene ontology enrichment analysis. In addition, comparing two biological replicates shows poor reproducibility between samples (Pearson’s r = 0.3), indicating that the few genes that show a change in expression are due to nonspecific effects of SPT inhibition (Fig. 3 b). These results revealed that despite a 40% drop in cellular levels of LCBs after 1 h of treatment, cells fail to trigger a specific transcriptional response. However, at the 3-h time point, almost the whole genome shows changes in transcript levels, and nearly every aspect of cell physiology seems to be affected as cells start to lose nuclear membrane integrity and viability (Fig. 3, b and c). Part of the changes at this point include a general response to stress, including the downregulation of ribosomal genes and the upregulation of several other processes, including protein degradation, autophagy, lipid metabolism, and vesicle transport (Fig. 3 c). These findings suggest that upon lowering sphingolipids, the alterations in gene expression may be an indirect response resulting from the loss of nuclear integrity and viability rather than the activation-specific signaling pathways.

### The biosynthesis of LCBs takes place upon entry into the cell cycle in yeast

**Figure 4.**
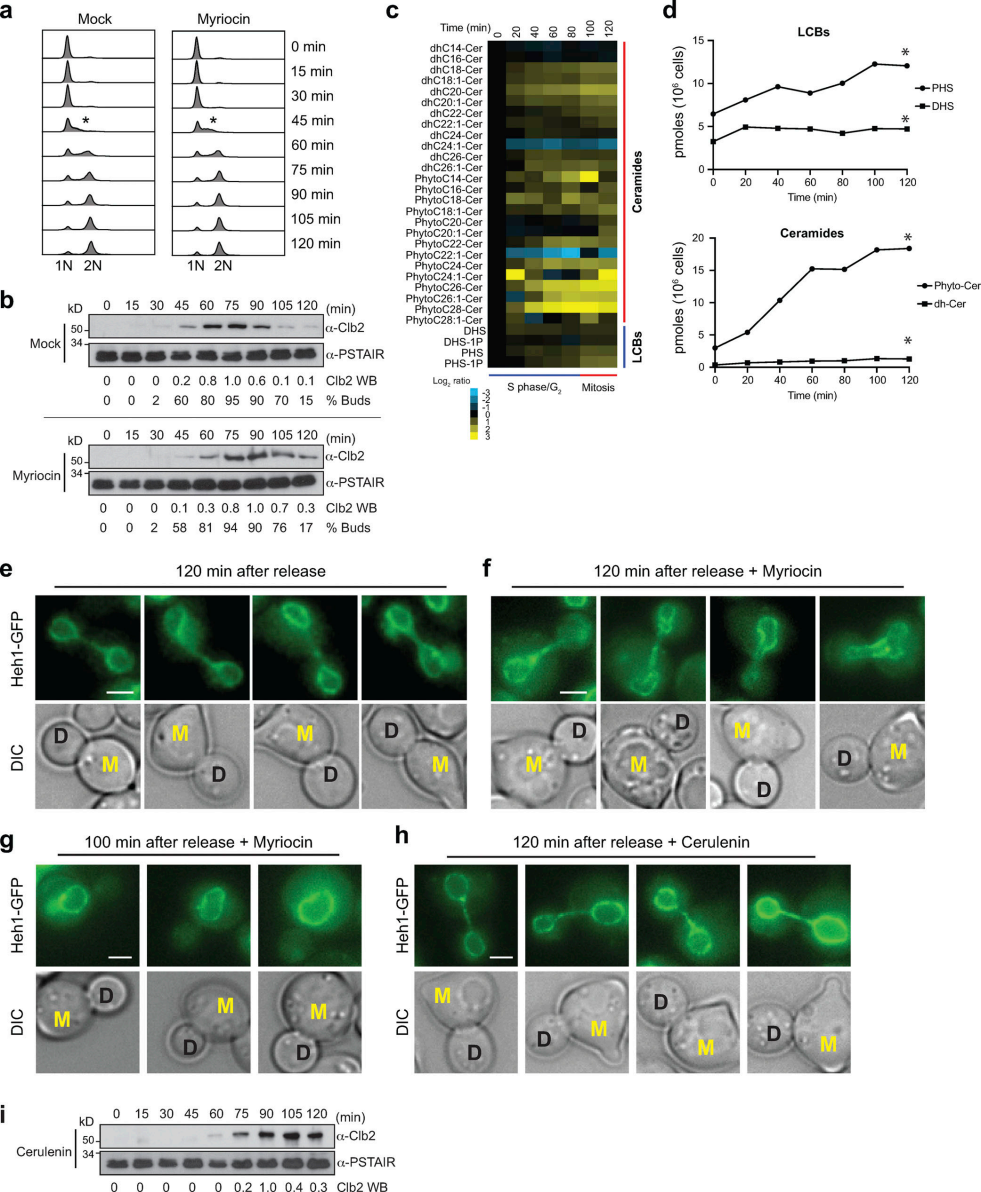
**LCB synthesis takes place upon entry into the cell cycle. (a)** DNA content of yeast cells (*n* = 30,000) after release from a pheromone-induced G1 arrest without or with myriocin (2.5 µM) at indicated time points. The asterisk denotes the beginning of the S phase. **(b)** Western blot analysis of mitotic Clb2 levels after release from G1 arrest. The PSTAIR antibody was used as a loading control. Relative levels of Clb2 quantified with ImageJ are shown under the western mages. The percentage of budded cells quantified by visual inspection of DIC images are shown below (*n* = 200). **(c)** HPLC-MS/MS analysis of LCB and ceramides in yeast cells harvested at indicated time points after release from a G1 arrest. Log_2_ ratios of the lipid levels relative to time 0 min are shown. Columns represent experiments, and rows represent lipid species. Cer, ceramide; dh, dihydro; DHS, dihydrosphingosine; PHS, phytosphingosine. **(d)** Plots of the total lipid classes as a function of time of the lipidome data presented in Fig. 2 c. *P < 1 e-40, paired *t* test comparison with time 0 min. **(e and f)** Live-cell microscopy of yeast cells expressing Heh1-GFP 120 min after the release from the G1 arrest in the minimal media without (e) or with (f) 2.5 µM myriocin. Scale bar, 2 µm. Mother cells show a shmoo from the G1 arrest and are labeled M. Daughter cells are labeled D. **(g)** Live-cell microscopy of yeast cells expressing Heh1-GFP 100 min after the release before anaphase from the G1 arrest in minimal media with 2.5 µM myriocin shows no defects. **(h)** Live-cell microscopy of yeast cells expressing Heh1-GFP 120 min after the release before anaphase from the G1 arrest in minimal media with 10 µM cerulenin. **(i)** Western blot analysis of mitotic Clb2 levels after release from G1 arrest in the presence of 10 µM cerulenin. The PSTAIR antibody was used as a loading control. Relative levels of Clb2 quantified with ImageJ are shown. HPLC-MS/MS, high-performance liquid chromatography–tandem mass spectrometry. Source data are available for this figure: SourceData F4.

To gain insight into the dynamics of the synthesis of LCBs as cells divide, we analyzed the cell cycle progression of yeast cells coupled with quantitative lipidomics. After the release from a G1 arrest in lipid-free media, yeast cells proceed synchronously through the cell cycle, completing DNA duplication within 75 min and mitosis after 2 h (Fig. 4, a and b). Quantitative lipidomics revealed that DHS and PHS increase during the first 20 min upon entry into the cell cycle (Fig. 4, c and d; and Table S3). Interestingly, after an initial rise, DHS does not further increase, while PHS continues to accumulate and precisely doubles from 6 to 12 pmol/10^6^ cells during G2 into mitosis about 100 min from the release. These results suggest that DHS conversion to PHS by the sphingosine hydroxylase *SUR2* occurs as cells enter mitosis. Consistent with this result, yeast cells do not accumulate dhCer; instead, they synthesize phytoceramides from PHS, which increased sixfold from 3 to 18 pmol/10^6^ cells before cytokinesis. Notably, a high accumulation of ceramides is observed in human cells at the end of mitosis, where ceramides are thought to play an essential role in cytokinesis (Atilla-Gokcumen et al., 2014).

Remarkably, myriocin treatment, which robustly inhibits the synthesis of DHS and PHS, fails to induce cell cycle arrest, and cells proceed normally into the S phase with unaffected DNA synthesis and bud formation (Fig. 4, a and b). In addition, the levels of mitotic cycling Clb2 are minimally affected by myriocin as cells show a slight delay in its synthesis. Still, degradation takes place with similar kinetics as in untreated cells (Fig. 4 b). While the nuclear morphology of myriocin-treated cells seems normal after cells are released from G1 and before they proceed into mitosis (Fig. 4 g), the integrity of the nuclear membrane is significantly compromised as the cells go through mitosis (Fig. 4, e and f). Analysis of the nuclear morphology of diving cells revealed that most daughter cells inherit an abnormal nucleus (Fig. 4 f). Nearly 100% of cells show abnormal nuclei during late anaphase and cytokinesis. Notably, within one cell cycle, cerulenin treatment does not cause defects in the morphology of the nucleus during anaphase and leads to the inhibition of Clb2 degradation at the end of mitosis (Fig. 4, h and i). These results indicate that sphingolipid synthesis occurs during the S and G2 phases of the cell cycle, coinciding with the time of DNA replication and increasing nuclear volume during cell division. If sphingolipid synthesis is inhibited, cells do not arrest at a particular cell cycle stage and proceed into mitosis. Unable to generate new membranes necessary to duplicate the nucleus, yeast cannot develop viable daughter cells.

### LCBs are integral components of the nuclear membrane in human cells

**Figure 5.**
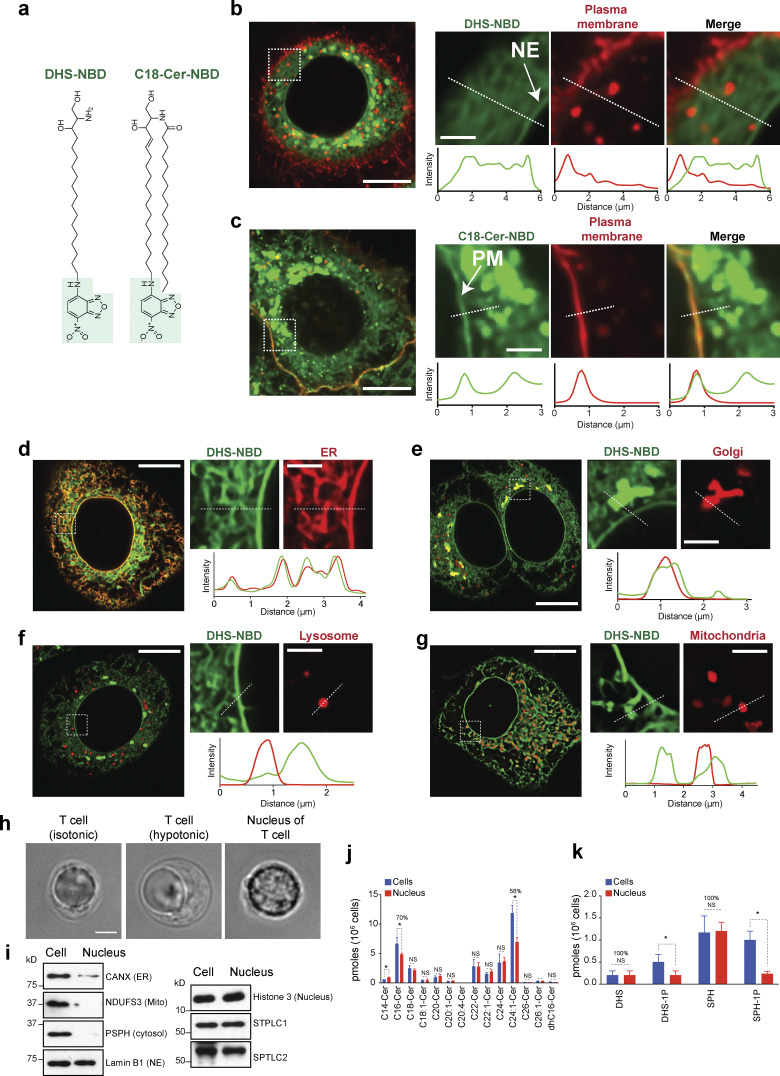
**LCBs are integral components of the nuclear membrane in human cells. (a)** Chemical structure of the fluorescent probes used in our studies. DHS, dihydrosphingosine; Cer, ceramide; NBD, nitrobenzoxadiazole. **(b)** Live-cell microscopy of HeLa cells expressing mCherry-Farnesyl-5 (red, plasma membrane) and incubated with 1 µM DHS-NBD (green) for 20 min. Scale bar in the left image, 10 µm, and in zoomed images, scale bar, 2 µm. NE indicates NE. Fluorescence intensity across the white dotted lines is shown below. **(c)** Live-cell microscopy of HeLa cells expressing mCherry-Farnesyl-5 and incubated with 1 µM C18-Cer-NBD (green) for 20 min. Scale bar in the left image, 10 µm; in zoomed images, scale bar, 2 µm. PM, plasma membrane. Fluorescence intensity across the white dotted lines is shown below. **(d–g)** Live-cell microscopy of HeLa cells incubated with 1 µM DHS-NBD (green) for 20 min. In d, cells express Sec61-mCherry (NE and ER); in e, cells express mCherry-Golgi-7 (Golgi apparatus); in f, cells express LAMP1-mCherry (lysosome); and in g, cell express mCherry-mito-7 (mitochondria). Scale bar in the left image, 10 µm; in zoomed images, scale bar, 2 µm. Fluorescence intensity across the white dotted lines is shown below. **(h)** DIC images of a T cell and purified nucleus. Scale bar, 2 µm. **(i)** Western blot analysis of the different organelle markers in whole-cell extracts and isolated nuclei from T cells. **(j and k)** HPLC-MS/MS analysis of ceramides (j) and LCBs (k) of primary T cells and isolated nuclei from the T cells. Error bars represent standard deviations (*n* = 3 independent samples from human blood, >10^6^ cells or nuclei analyzed per sample). *P < 1e-4, unpaired *t* test. NS, P value >0.05. HPLC-MS/MS, high-performance liquid chromatography–tandem mass spectrometry. Source data are available for this figure: SourceData F5.

**Figure S2.**
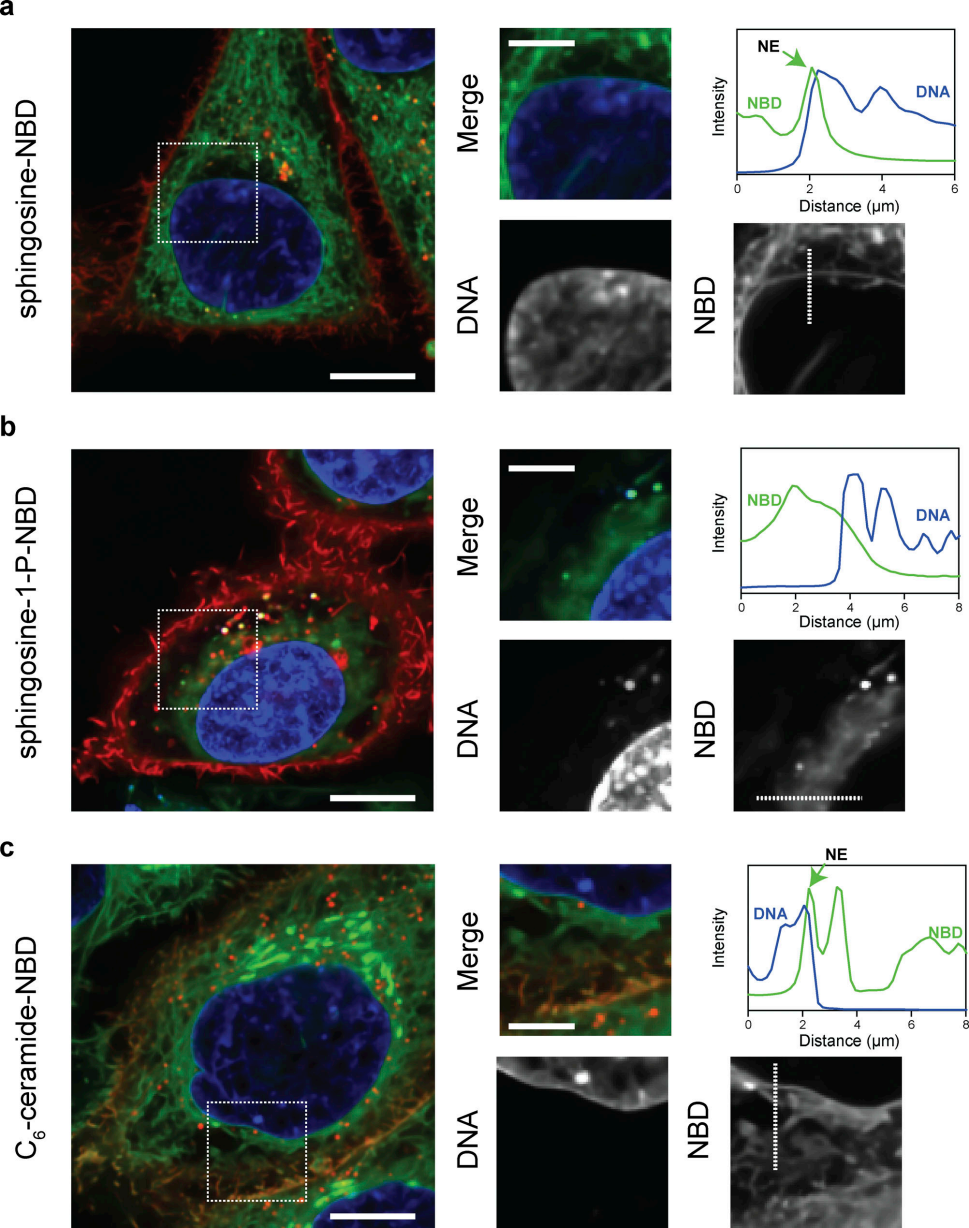
**LCBs are integral components of the nuclear membrane in human cells. (a–c)** Live-cell microscopy of HeLa cells expressing mCherry-Farnesyl-5 (red, plasma membrane) incubated for 20 min with 1 µM Sph-NBD (a), S1P-NBD (b), or C6-ceramide-NBD (c). Scale bar, 5 µm. Scale bar in zoomed images, 1 µm.

Next, we investigated whether the role of the synthesis of LCBs in supporting nuclear membrane integrity in yeast is conserved in human cells. To gain insight into the localization of LCBs in human cells, we used a DHS molecule labeled with nitrobenzoxadiazole (NBD, Fig. 5 a). Live-cell microscopy of HeLa cells incubated with DHS-NBD for 20 min shows that this lipid molecule gets internalized and localizes in several intracellular membranes, including the NE (Fig. 5, b and d). Interestingly, C18-ceramide-NBD also gets internalized within 20 min but does not localize to the NE (Fig. 5 c). Another striking difference is that C18-ceramide-NBD accumulates at the plasma membrane, while DHS-NBD does not (Fig. 5, b and c). Despite the nonspecific diffusion of exogenous lipids throughout the cell, these data are consistent with the hypothesis that LCBs preferentially localize in internal membranes, including the NE/ER compartment of the cell, while ceramide can freely diffuse into the plasma membrane. Notably, we could not detect differences in the cellular localization of DHS-NBD and Sph-NBD (Fig. S2 a). However, S1P-NBD is internalized less efficiently and does not label internal membranes compared with DHS- or Sph-NBD (Fig. S2 b). Meanwhile, C6-ceramide-NBD is very toxic to the cell, often causing abnormal nuclear shape and labeling of both the plasma membrane and NE. Our data indicate that C6-ceramide does not mimic the impact of C18-ceramide or LCBs on the cell (Fig. S2 c).

We then analyzed DHS-NBD localization in HeLa cells expressing fluorescent probes that label different organelles. Live-cell microscopy revealed that DHS-NBD colocalizes with mCherry-Sec61 in the NE/ER (Fig. 5 d). In addition, DHS-NBD colocalizes with mCherry-Golgi-7 (N-terminal domain of B4GALT1) at the Golgi apparatus, which is closely associated with the nucleus (Fig. 5 e). However, DHS-NBD does not colocalize with lysosomal LAMP1-mCherry (Fig. 5 f) or mCherry-mito-7 (mito-COX8A, Fig. 5 g). These data show that although it can freely diffuse throughout the cell, DHS preferentially integrates into specific organelles, indicating that the lipid composition and biochemical properties of different compartments determine the affinity for exogenous DHS molecules. Together, these data support the hypothesis that SPT makes LCBs, which remains localized in the NE/ER and Golgi apparatus and is minimally incorporated into other organelles. Notably, the usefulness of these probes to visualize lipids in vivo is limited because they are toxic to the cell, as longer incubations showed drastic effects on the organization of the cytosol and lowered viability.

To examine the localization of endogenous LCBs, we attempted to purify nuclei of several cell lines, including untransformed retinal pigmental epithelial cells (RPE-1 hTERT, thereafter RPE-1) and HeLa cells. However, given that the ER is continuous with the NE/ER is in physical contact with other organelles, including the plasma membrane and mitochondria, without mild detergents, we failed to generate nuclear preparations that were pure enough to determine nuclear lipid composition. Therefore, we turned to primary T cells because these cells have a small cytoplasm and can be easily ruptured by hypotonic treatment. Using western blot analysis and visual inspection by differential interference contrast (DIC) microscopy, nuclear preparations from primary T cells show little contamination from other organelles (Fig. 5, h and i). Quantitative lipidomics of T cells from three independent donors revealed that nearly 100% of total LCBs in the cell are in the nuclear fractions, while only 58% of C24:1-ceramide and 70% of C16-ceramide, the most abundant ceramide species, are in the nucleus (Fig. 5, j and k). These results are consistent with the lipidomics analysis in yeast, indicating that LCBs are primarily concentrated in the nuclear membrane of the cell.

### Inhibition of SPT disrupts nuclear morphology in human cells

**Figure 6.**
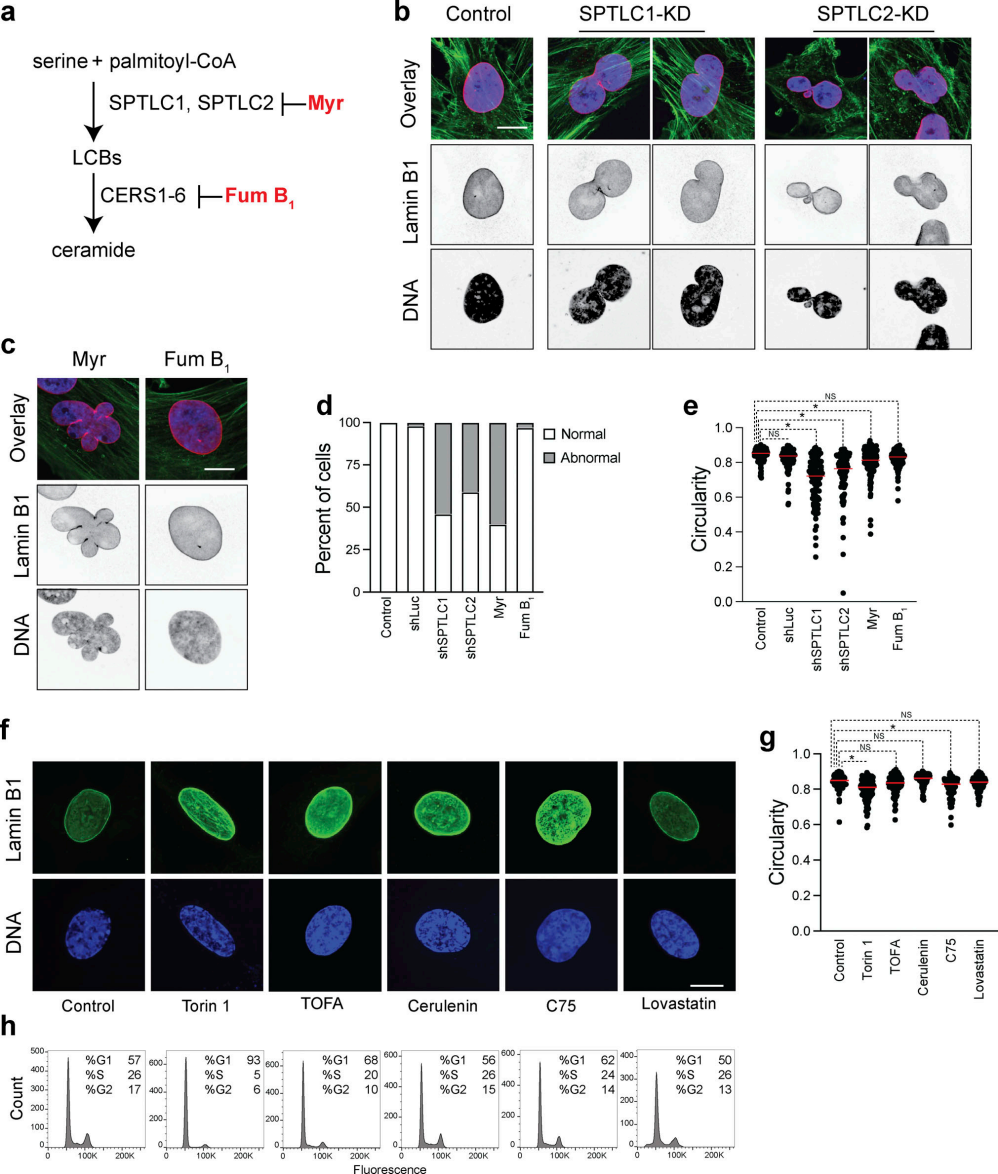
**Inhibition of SPT disrupts nuclear morphology in human cells. (a)** *De novo* synthesis pathway of sphingolipids in humans. See Fig. S1 b for a more detailed description of the pathway. Chemicals used in our experiments are highlighted in red: Myr, myriocin; Fum B_1_, fumonisin B_1_. SPTC1 and SPTLC2 are the main subunits of SPT; LCBs, long-chain bases; CerS, ceramide synthase. **(b)** Representative immunofluorescence images of RPE-1 cells upon knockdown of scramble sequence (Control), SPTLC1, or SPTLC2. Green, anti-alpha-tubulin; purple, Hoechst 33342 (DNA); and red, anti-lamin B1. Scale bar, 5 µm. Some images are also shown in Fig. S4 c. **(c)** Representative images of cells treated with 5 µM myriocin or 10 µM fumonisin B1. Immunofluorescence markers as in b. Scale bar, 5 µm. Some images are also shown in Fig. S4 c. **(d)** Percentage of the nuclear phenotype of RPE-1 cells. Cells were treated with 5 µM myriocin or 10 µM fumonisin B1 (*n* = 200 cells). Abnormal refers to irregular nuclear shape relative to normal round nuclei. **(e)** Circularity (ImageJ) of 200 nuclei for each condition. *P < 1e-4, one-way ANOVA. **(f)** Representative images of RPE-1 cells in lipid-depleted media treated with 250 nM Torin, 10 µM TOFA, 10 µM cerulenin, 40 µM C75, or 1 µM lovastatin cells for 24 h. Blue, Hoechst 33342 (DNA); green, anti-lamin B1 (NE). Scale bar, 5 µm. **(g)** Circularity (ImageJ) of 200 nuclei for each condition in Fig. 5 f. *P < 1e-4, one-way ANOVA. **(h)** FACS profiles of cells in each condition in Fig. 5 f.

**Figure S3.**
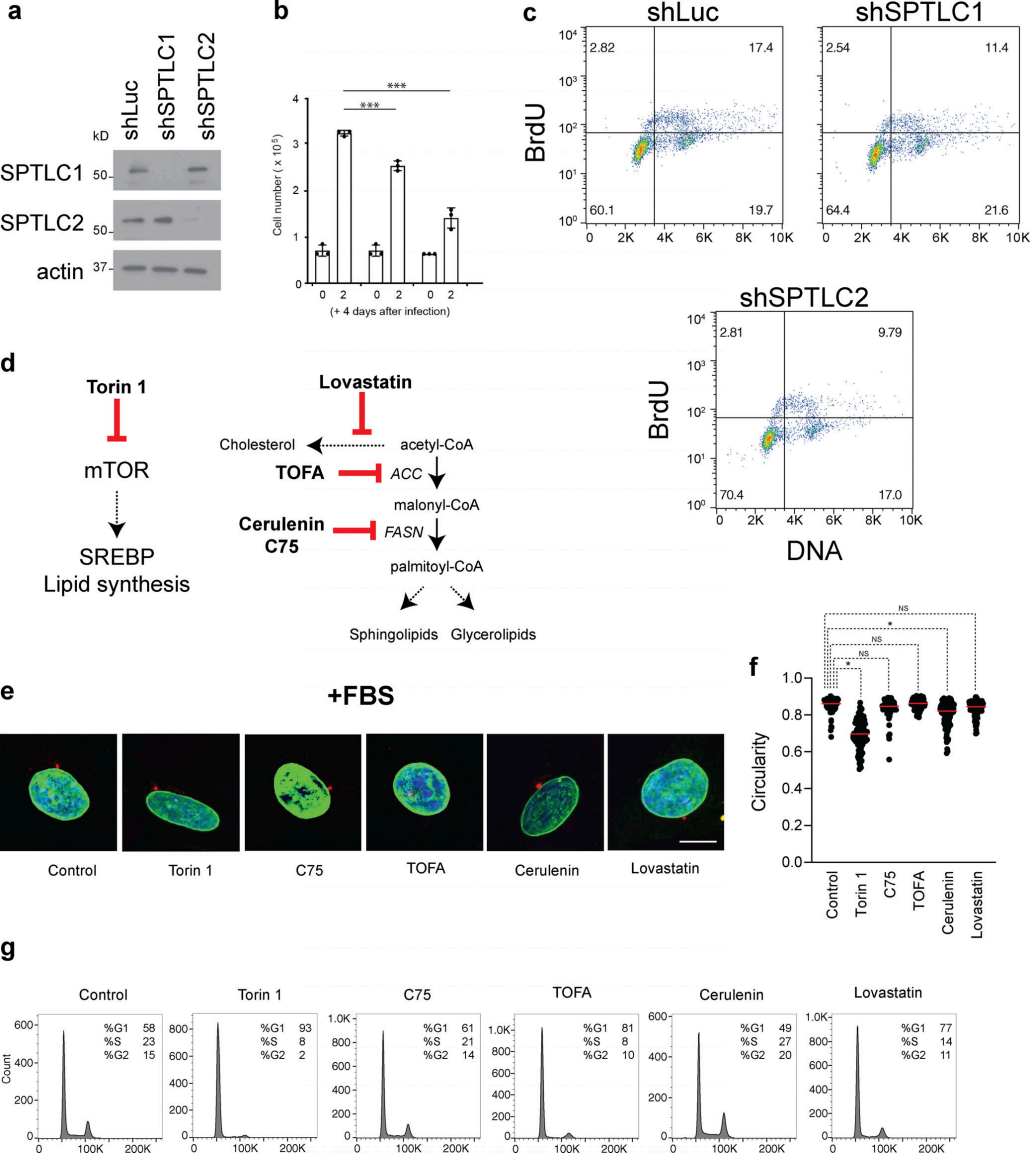
**Inhibition of SPT disrupts nuclear morphology in human cells. (a)** Western blot of SPT subunits in RPE-1 upon their knockdown for 48 h. **(b)** Cell proliferation of RPE-1 upon knockdown of SPTLC1 or SPTLC2. Error bars represent standard deviations (*n* = 3). *P < 1e-4, one-way ANOVA. **(c)** FACS profiles of cells stained with BrdU upon knockdown of SPTLC1 or SPTLC2. **(d)** Schematic of target enzymes by the different drugs used in our experiments. **(e)** Representative images of RPE-1 cells in media containing 10% FBS in the presence of 250 nM Torin, 10 µM TOFA, 10 µM cerulenin, 40 µM C75, or 1 µM lovastatin. Blue, Hoechst 33342 (DNA); green, anti-lamin B1 (NE). Scale bar, 5 µm. **(f)** Circularity of 200 nuclei for each condition in Fig. S3 e. **(g)** FACS profiles of cells in each condition in Fig. S3 e. Source data are available for this figure: SourceData FS3.

To investigate the physiological consequences of lowering the levels of LCBs, we used immunofluorescence to determine the shape and integrity of the nucleus in RPE-1 cells upon knockdown of either subunit of SPT (*SPTLC1* or *SPTLC2*, Fig. 6 a and Fig. S3 a). Consistent with previous results, we found that after 48 h, the knockdown of *SPTLC1* or *SPTLC2* disrupts the nuclear morphology of RPE-1 cells (Fig. 6, b and d). Notably, SPT knockdown hampers proliferation in a medium containing lipids (10% fetal bovine serum, FBS), but we could not detect signs of cell cycle arrest or cell death (Fig. S3, b and c). However, cells did not survive SPT knockdown in media depleted of lipids (lipid-depleted FBS, LD-FBS), indicating that SPT downregulation can be better tolerated in the presence of exogenous lipids. Indeed, we found that chemical inhibition of SPT with myriocin for 24 h in LD-FBS causes similar phenotypes as found upon genetic disruption (Fig. 6, c and d), while inhibition of ceramide synthesis using fumonisin B_1_ does not affect the morphology of the nucleus (Fig. 6, c and d). Fumonisin B_1_ causes the accumulation of LCBs while lowering ceramide levels and does not affect proliferation (Hwang et al., 2019). Analysis of shape parameters indicates that targeting SPT activity, genetically or chemically, affects the circularity of the nucleus compared with fumonisin B_1_ treatment or the control condition (Fig. 6 e). These results show that lowering LCBs and not ceramides causes nuclear abnormalities in human cells.

Next, we targeted other pathways associated with lipid synthesis to investigate whether lowering other lipids affects nuclear morphology. We found that the integrity of the nucleus of RPE-1 cells is unaffected upon treatment with the inhibitor of mTOR Torin 1, the FAS inhibitors cerulenin or C75, the ACC inhibitor TOFA, or the HMG-CoA reductase inhibitor lovastatin, which reduces cholesterol (Fig. 6, f and g; and Fig. S3 d). The nuclear shape becomes more elongated upon mTOR inhibition without affecting the integrity of the nuclear membrane, in agreement with previous reports (Peterson et al., 2011). In addition, the effects of these five drugs are the same when cells are grown in a medium containing FBS or LD-FBS (Fig. 6, f and g; and Fig. S3, e and f). Lastly, it is notable that treatment with these drugs affects cell proliferation and does not cause a significant accumulation of cells at a particular cell cycle stage, except mTOR inhibition, which causes cell cycle arrest in G1 (Fig. 6 h and Fig. S3 g). These results indicate that disruption of the nuclear morphology is specific to inhibiting the synthesis of LCBs and that inhibition of lipid synthesis, in general, does not cause nuclear abnormalities.

### The biosynthesis of LCBs takes place upon entry into the cell cycle in human cells

**Figure 7.**
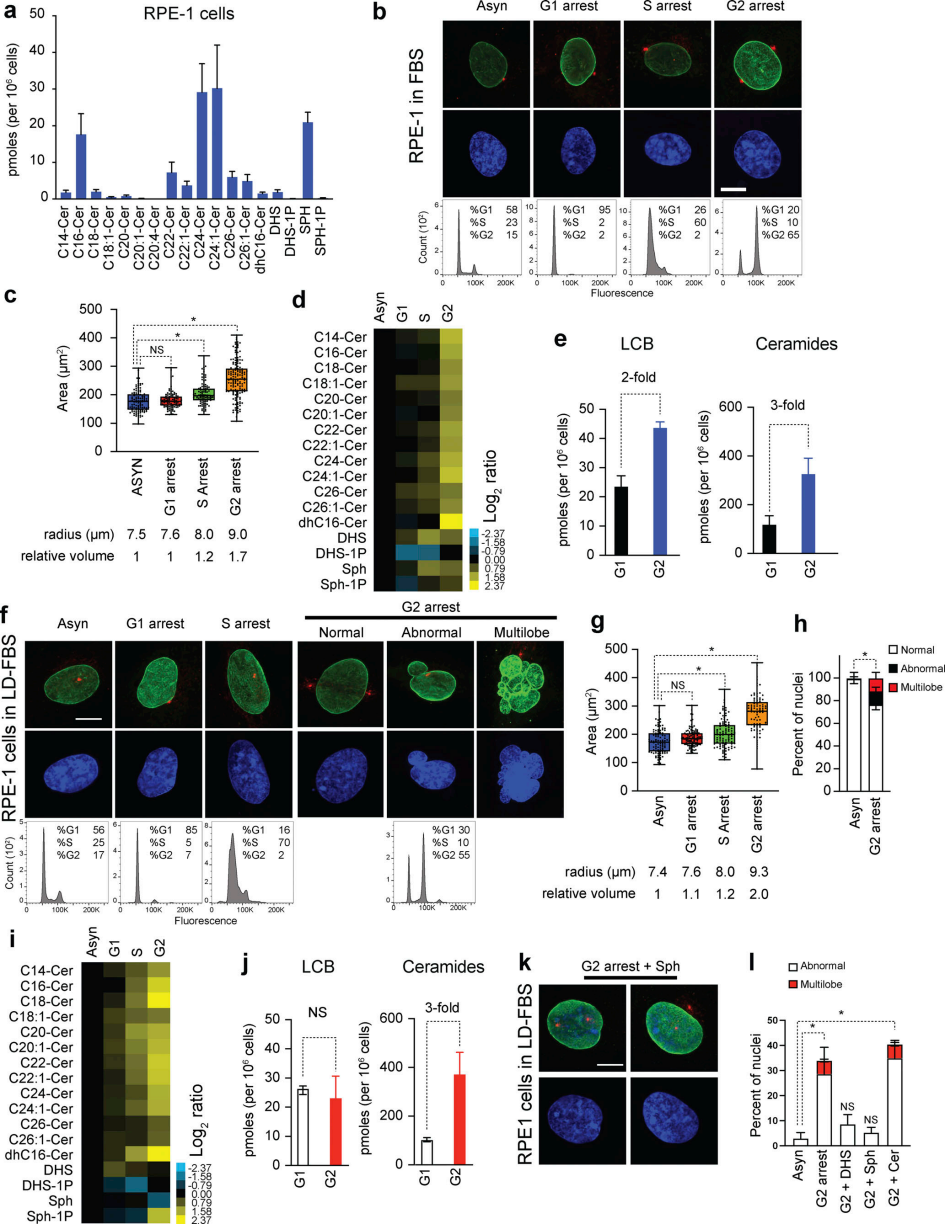
**LCBs are synthesized during S and G2 phases in human cells. (a)** HPLC-MS/MS analysis of LCBs and ceramides of RPE-1 cell culture in 10% FBS. Error bars represent the standard deviation (*n* = 3 biological replicates, >10^6^ cells analyzed in each experiment). **(b)** Representative images of RPE-1 cells in 10% FBS (Asynchronous, Asyn) or arrested in G1 with 2 µM palbociclib, in the S phase with 2 µM thymidine, or in late G2 with 10 µM RO-3306. Blue, Hoechst 33342 (DNA); and green, anti-lamin B1. Scale bar, 5 µm. FACS profiles are shown below. **(c)** Area of nuclei of RPE-1 cells (*n* = 200) as in b. The averaged calculated radius is shown below. *P < 1e-4, one-way ANOVA. **(d)** HPLC-MS/MS analysis of LCBs and ceramides in RPE-1 arrested as in b. Columns represent experiments (*n* = 3 biological replicates, >10^6^ cells analyzed in each experiment), and rows represent lipid species. Cer, ceramide; dh, dihydro; DHS, dihydrosphingosine; Sph, sphingosine. **(e)** Average log_2_ ratios of the lipid levels in G1- or G2-arrested cells are shown. Error bars represent standard deviations (*n* = 3 biological replicates). **(f)** Representative images of RPE-1 cells in LD-FBS (Asynchronous, Asyn) or arrested in G1 with 2 µM palbociclib, in the S phase with 2 µM thymidine, or in late G2 with 10 µM RO-3306. Blue, Hoechst 33342 (DNA); and green, anti-lamin B1. Scale bar, 5 µm. FACS profiles are shown below. **(g)** Area of nuclei of RPE-1 cells (*n* = 200 nuclei) as in f. The averaged calculated radius is shown below. Nuclei with abnormal morphology are not included in G2 arrest. *P < 1e-4, one-way ANOVA. **(h)** Percentage of cells with abnormal nuclear morphology. Error bars represent the standard deviation of 3 biological replicates (each replicate, *n* >100 cells). *P < 1e-4, unpaired *t* test. **(i)** HPLC-MS/MS analysis of LCBs and ceramides in RPE-1 arrested as in f. Log_2_ ratios of the lipid levels relative to asynchronous cells are shown. Columns represent experiments, and rows represent lipid species. Cer, ceramide; dh, dihydro; DHS, dihydrosphingosine; Sph, sphingosine. Scale bar, 5 µm. **(j)** Quantification of total LCBs and ceramides in G1- or G2-arrested RPE-1 cells in LD-FBS. Error bars represent the standard deviations (*n* = 3 biological replicates). **(k)** Representative images of RPE-1 cells in lipid-depleted media arrested in G2 in the presence of 1 µM Sph. Blue, Hoechst 33342 (DNA); and green, anti-lamin B1. Scale bar, 5 µm. **(l)** Percentage of cells with abnormal nuclear morphology. Error bars represent the standard deviation of three independent biological replicates (each replicate, *n* > 100 cells). Cells were arrested in G2 alone or with 1 µM DHS, 1 µM Sph, or 1 µM ceramide. *P < 1e-4, one-way ANOVA. HPLC-MS/MS, high-performance liquid chromatography–tandem mass spectrometry.

**Figure S4.**
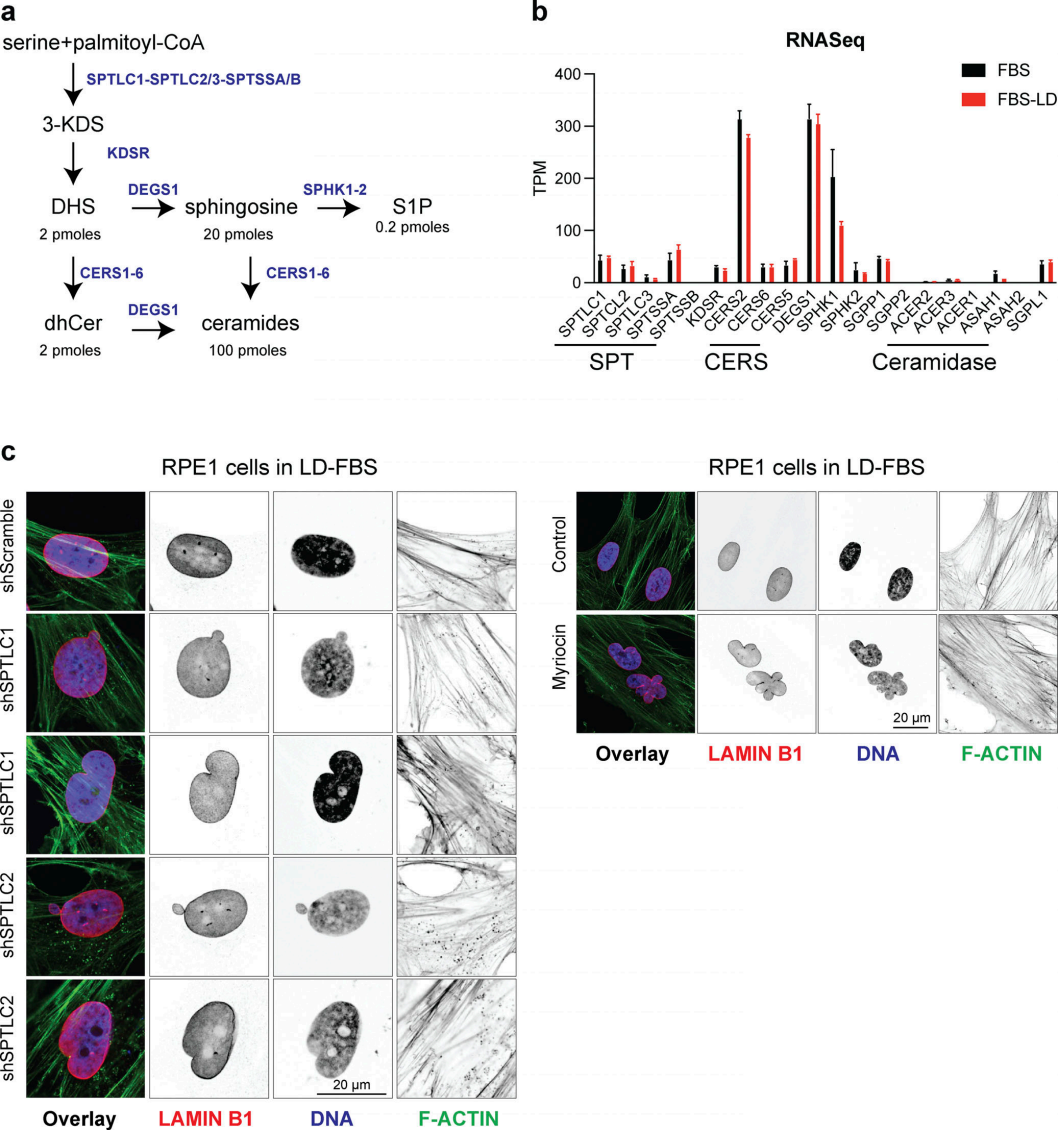
**LCBs are synthesized during S and G2 phases in human cells. (a)** Sphingolipid synthesis pathway in human cells showing the levels of lipid molecules measured by mass spectroscopy in 106 cells. **(b)** Gene expression of the enzymes involved in de novo synthesis of sphingolipids in RPE-1 cells. **(c)** Representative immunofluorescence images of RPE-1 cells upon knockdown of scramble sequence (Control), SPTLC1, or SPTLC2. Green, phalloidin staining of f-actin; purple, Hoechst 33342 (DNA); and red anti-lamin B1.

Quantitative lipidomics revealed that on average, RPE-1 cells consist of 2 pmol/10^6^ cells of DHS, a 10-fold excess of Sph (20 pmol/10^6^ cells), and much lower levels of phosphorylated Sph (0.2 pmol/10^6^ cells, Fig. 7 a, Fig. S4 a, and Table S4). Ceramides are close to fivefold the levels of Sph (100 pmol/10^6^ cells). The chain lengths of the second FA added to Sph to generate ceramides are mainly 24, 16, and 22 carbons (C24 > C16 > C22). Interestingly, RNA-seq shows these cells express CerS2 and lower levels of CerS5 and CerS6, supporting the hypothesis that these isoforms have specificity for acyl chains that consist of 24, 22, and 16 carbons (Levy and Futerman, 2010) (Fig. S4 b and Table S5). Noteworthily, total levels of dhCer are close to 2 pmol/10^6^ cells, indicating a high rate of the conversion of dhCer to ceramides driven by the delta 4-desaturase, sphingolipid 1 *DEGS1* (Ternes et al., 2002). Indeed, *DEGS1* is among the highly expressed genes in RPE-1 cells relative to the other enzymes in the de novo synthesis pathway of sphingolipids (Fig. S4 b).

To investigate how the levels of LCBs change during cell division in human cells, we performed quantitative lipidomics at various cell cycle stages (Fig. 7, b–e). First, to determine whether LCBs or ceramides accumulate during G1, we treated cells for 24 h with palbociclib, a Cdk4/6 inhibitor. RPE-1 cells in G1 show a round nucleus (circularity ∼0.9), one centrosome juxtaposed to the NE, and an averaged nuclear volume of 1.8 pL (r = 7.6 µm), similar to the average nuclear volume of the asynchronous population (r = 7.5 µm). Quantitative lipidomics shows no significant changes in the levels of LCBs or ceramides during the G1 arrest (Fig. 7 d).

Next, we treated cells with thymidine for 24 h, arresting cells throughout the S phase. At this stage, cells show a round nucleus with a slight increase in nuclear volume (2.1 pL, r = 8 µm) compared with G1-arrested cells (Fig. 7 c). The levels of LCBs almost doubled from 20 to 35 pmol/10^6^ cells in the S phase, while ceramides increased 1.3-fold relative to G1 (Fig. 7 d). Consistent with the measurements in yeast, these results indicate that de novo synthesis of sphingolipids occurs during the S phase of the cell cycle. Lastly, we treated cells with RO-3306, a drug that inhibits Cdk1 and arrests cells in late G2. Here, cells show duplicated centrosomes in opposite poles of a round nucleus with a 1.7-fold increase in volume (3 pL, r = 9.0 µm) relative to G1 cells (Fig. 7, b and c). At this point, the levels of LCBs doubled to 40 pmol/10^6^ cells, and ceramides increased close to threefold to 320 pmol/10^6^ cells (Fig. 7, d and e). Notably, G1-, S-, or G2-arrested cells show a round nucleus with centrosomes juxtaposed to the NE, suggesting a physical interaction between these organelles (Bolhy et al., 2011; Bornens, 1977). These results show that the levels of LCBs and ceramides increase during the S and G2 phases of the cell cycle and that these increases correlate with increases in nuclear volume.

Next, we tested whether exogenous lipids influence cell cycle–dependent changes in lipid levels and nuclear volume. We found that cells arrested in G1 in lipid-depleted media show similar nuclear shapes, nuclear volumes, and lipid levels compared with cells in G1 in FBS (Fig. 7, f and g). As expected, cells arrested in the S phase show normal nuclear morphology and a slight increase in nuclear volume compared with G1 cells. However, LCB levels unexpectedly did not change in the S phase, while ceramide levels increased 1.3-fold (Fig. 7 i). Indeed, when cells were arrested in G2 in lipid-depleted media, LCB levels did not significantly change relative to G1-arrested cells, while ceramides still increased an average of threefold (Fig. 7 j). Despite LCB levels not increasing during the G2 arrest, the nuclear volume almost doubled compared with G1-arrested cells from 1.8 to 3.4 pL. However, we found that G2-arrested cells show nuclear morphologies severely compromised. While most cells show typical round morphology and duplicated centrosomes, about one third showed abnormal shapes relative to controls. In addition, ∼10% of cells showed a drastic disruption of nuclear integrity and morphology, reminiscent of multilobed nuclei (Fig. 7, f and h). These results indicate that the failure to increase LCBs during the S and G2 phases profoundly impacts nuclear integrity. Moreover, our results suggest that the increases of the levels of LCBs during the cell cycle are Cdk1-dependent, and exogenous lipids in the FBS medium suppress nuclear defects upon Cdk1 inhibition. Indeed, we found that adding exogenous DHS or Sph alone, and not ceramides, suppressed nuclear abnormalities of cells arrested in G2 in the lipid-depleted medium (Fig. 7, k and l). Our findings support the hypothesis that increasing the levels of LCBs is crucial for expanding nuclear volumes during the cell cycle.

### Abnormal nuclear morphology upon SPT inhibition arises following cell division

**Figure 8.**
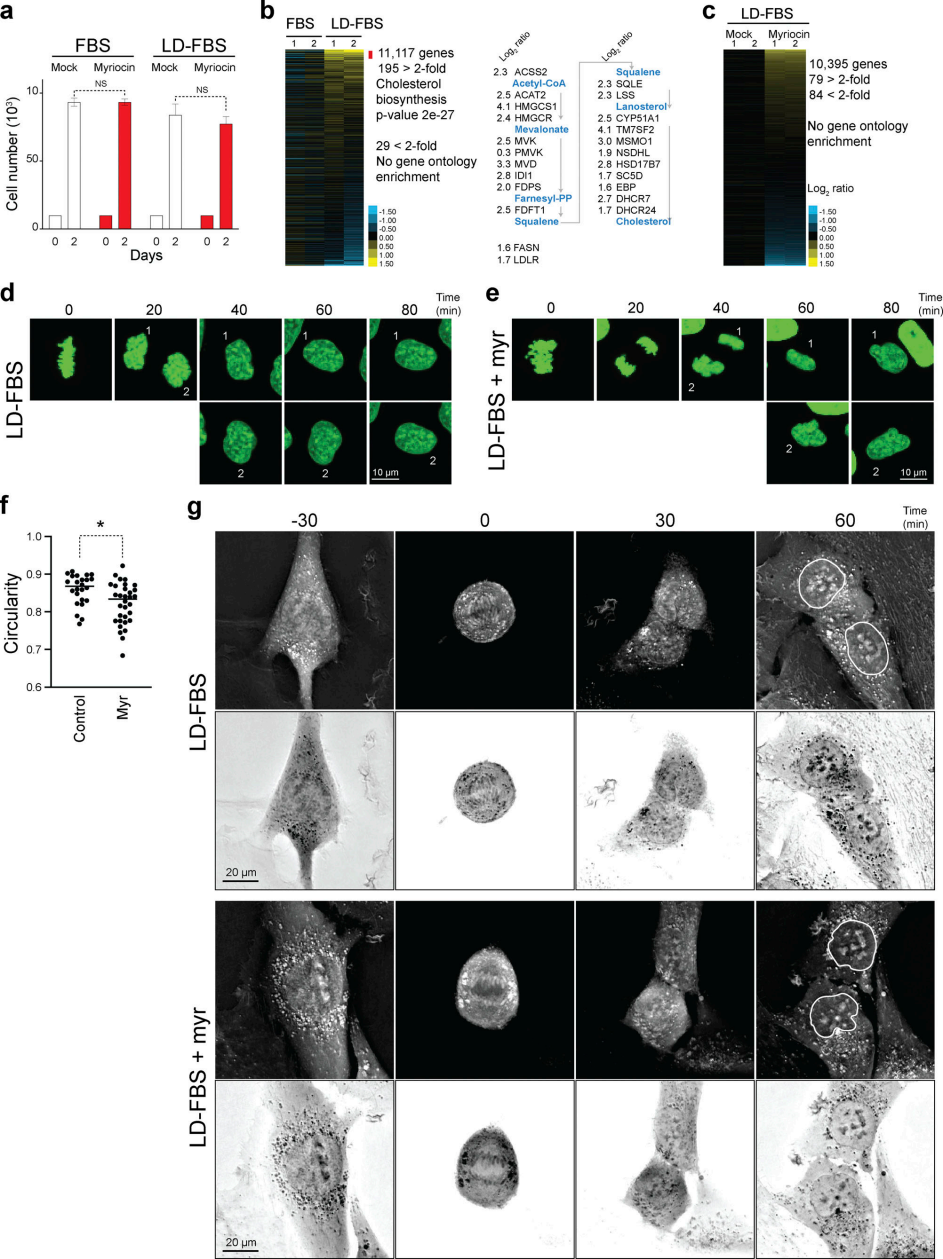
**Abnormal nuclear morphology upon SPT inhibition arises following cell division. (a)** Proliferation of RPE-1 cells in the medium with 10% FBS or LD-FBS without or with 5 µM myriocin for 48 h. Cell numbers were quantified with a Coulter counter. Error bars represent the standard deviation (*n* = 3 biological replicates). NS, P > 0.05. **(b)** Transcriptome profile of RPE-1 cell culture in 10% FBS or LD-FBS. The number of genes upregulated in LD-FBS compared with FBS media includes every enzyme in the cholesterol biosynthesis pathway. **(c)** Transcriptome profile of RPE-1 cells cultured in LD-FBS without and with 5 µM myriocin for 24 h. No significant enrichment of gene ontology terms found in the genes up- or downregulated. **(d)** Time lapse of live-cell microscopy images of RPE-1 expressing GFP-histone H2B in LD-FBS. Scale bar = 10 µm. **(e)** Time lapse of live-cell microscopy images of RPE-1 expressing GFP-histone H2B in LD-FBS in the presence of 5 µM myriocin for 20 h. Scale bar = 10 µm. **(f)** Quantification of the circularity of the nuclei of daughter cells 2 h after metaphase was observed in control cells or cells treated with 5 µM myriocin for 20 h. See Videos 1 and 2. *n* > 30, *P < 0.05, unpaired *t* test. **(g)** Time-lapse images of RPE-1 cells using a Nanolive microscope in LD-FBS without and with 5 µM myriocin for 20 h. The nuclear shape of the daughter cell is highlighted in the 60-min time point by a white line contour. Scale bar = 20 µm. See Videos 3 and 4.

To gain insight into how the lowering of LCBs disrupts the morphology of the nucleus, we characterized cells grown in LD-FBS treated with myriocin for 24 h when the abnormal nuclear phenotype starts to appear. We found that culturing cells in LD-FBS show similar proliferation rates to those in FBS (Fig. 8 a). Transcriptome analysis indicates that the gene expression of enzymes in the de novo synthesis of sphingolipid pathway is not affected when lipids are depleted from the growth medium (Fig. S4, a and b). Instead, cells upregulate genes in the cholesterol biosynthesis pathway, FAS, and the LDL receptor (Fig. 8 b and Table S5). Furthermore, myriocin treatment does not significantly affect proliferation rates in either FBS or LD-FBS, and as observed in yeast, myriocin treatment for one doubling time (24 h) in LD-FBS elicits no significant changes in gene expression (Fig. 8, a and c). Remarkably, live-cell microscopy of RPE-1 cells expressing histone H2B tagged with GFP shows that newly formed nuclei display abnormal morphology in the presence of myriocin compared with untreated controls after mitosis (Fig. 8, d and e; and Videos 1 and 2). Analysis of the shape parameters of newly formed nuclei 2 h after metaphase shows that the circularity of the nucleus is affected upon SPT inhibition (Fig. 8 f). To visualize the NE directly, we used a label-free live-cell imaging technique that uses the refractive index to visualize cell structures. Consistently, the NE of newly formed cells in the presence of myriocin shows abnormal morphologies compared with untreated controls (Fig. 8 g; and Videos 3 and 4). Together, these results indicate that as observed in yeast, lowering LCBs does not interfere with cell cycle progression or elicit significant changes in global gene expression, yet inhibiting LCB synthesis disrupts the formation of the new nucleus of the daughter cell after mitosis.

**Video 1.** **Live time-lapse fluorescence microscopy of RPE-1 expressing GFP-histone H2B in LD-FBS without any drugs.** Green signal from GFP was visualized in frames every 20 min at a rate of 1 frame per sec. Related to Fig. 8 d.

**Video 2.** **Live time-lapse fluorescence microscopy of RPE-1 expressing GFP-histone H2B in LD-FBS with 5 µM myriocin.** Green signal from GFP was visualized in frames every 20 min at a rate of 1 frame per sec. Related to Fig. 8 e.

**Video 3.** **Nanolive live-cell microscopy of RPE-1 cells in LD-FBS.** Images are generated from the refraction index in frames every 5 min at a rate of 1 frame per sec. Related to Fig. 8 g.

**Video 4.** **Nanolive live-cell microscopy of RPE-1 cells in LD-FBS with 5 µM myriocin.** Images are generated from the refraction index in frames every 5 min at a rate of 1 frame per sec. Related to Fig. 8 g.

### Lack of LCBs causes genomic instability

**Figure 9.**
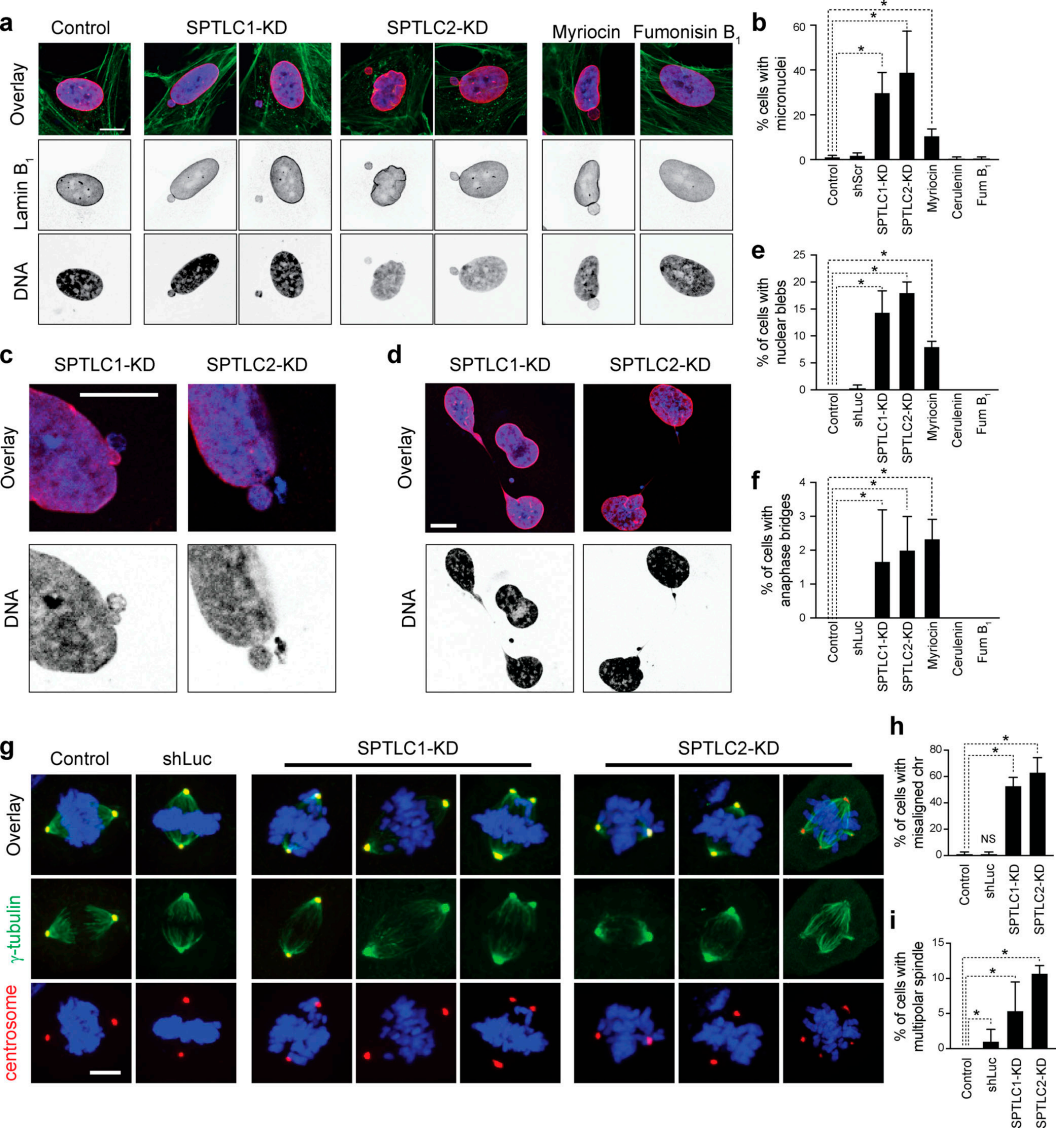
**Lack of LCBs causes genomic instability. (a)** Representative immunofluorescence images of RPE-1 cells upon knockdown of scramble (Control), *SPTLC1*, or *SPTLC2* cultured for 96 h, or treated with 5 µM myriocin or 10 µM fumonisin B_1_ in lipid-depleted medium for 48 h. Green, anti-alpha-tubulin; purple, Hoechst 33342 (DNA); and red, anti-lamin B1. Scale bar, 5 µm. Some images are also shown in Fig. S4 c. **(b)** Quantification of cells with micronuclei. Error bars represent the standard deviation of three biological replicates (each replicate, *n* > 100 cells). *P < 1e-4, one-way ANOVA. **(c)** Representative immunofluorescence image of RPE-1 nuclei showing NE blebs upon knockdown of *SPTLC1* or *SPTLC2*. Scale bar, 5 µm. **(d)** Representative immunofluorescence image of RPE-1 cells with an anaphase bridge upon knockdown of *SPTLC1* or *SPTLC2*. Scale bar, 5 µm. **(e)** Quantification of cells with nuclear blebs. Error bars represent the standard deviation of three biological replicates (each replicate, *n* > 100 cells). *P < 1e-4, one-way ANOVA. **(f)** Quantification of cells with anaphase bridge. Error bars represent the standard deviation of 3 biological replicates (each replicate, *n* > 100 cells). *P < 1e-4, one-way ANOVA. **(g)** Representative immunofluorescence images of RPE-1 cells 45 min after release from a G2 arrest. Green, anti-gamma-tubulin; blue, Hoechst 33342 (DNA); and red, anti-CDK5RAP2 (centrosome). Scale bar, 5 µm. **(h and i)** Quantification of cells with misaligned chromosomes (h) or multipolar spindle (i). Error bars represent the standard deviation of three biological replicates (each replicate, *n* > 100 cells). *P < 1e-3, one-way ANOVA.

**Figure S5.**
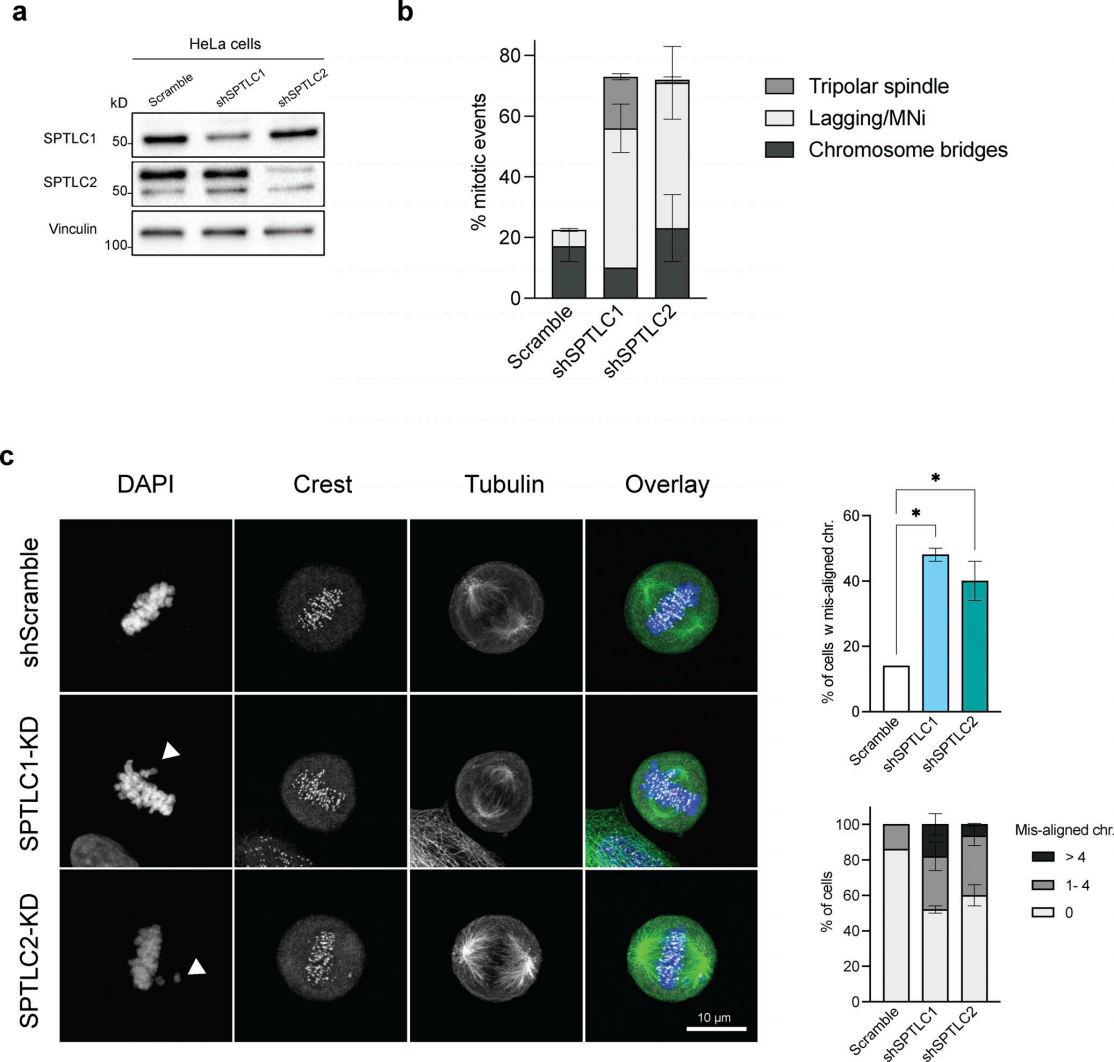
**Lack of LCB causes genomic instability. (a)** Western blot of SPT subunits in HeLa cells upon their knockdown. **(b)** Quantification of mitotic events in HeLa cells upon SPTLC1 or SPTLC2 knockdowns. Error bars represent the standard deviation of three biological replicates. **(c)** Representative images highlighting chromosome missegregation events in HeLa cells upon knockdown of SPTLC1 or SPTLC2. Quantification of the phenotypes is shown on the right. Source data are available for this figure: SourceData FS5.

48 h after SPT knockdown or 24 h after myriocin treatment, RPE-1 cells do not arrest in the cell cycle or show signs of cell death (Fig. S3 c). Therefore, to characterize the longer term consequences of inhibiting the biosynthesis of LCBs, we analyzed the nuclear morphology of cells cultured for 4 days after SPT knockdown or 2 days after myriocin treatment. Strikingly, we found that either approach to lowering the levels of LCBs increases the incidence of micronuclei and causes the appearance of nuclear blebs and, at low incidence, anaphase bridges (Fig. 9, a–f). Importantly, neither inhibition of ceramide synthesis by fumonisin B1 nor FAS inhibition by cerulenin causes similar phenotypes or shows any signs of genomic instability (Fig. 9, a, b, e, and f). We also found that SPT inhibition increases the number of micronuclei, mitotic errors, and multipolar mitosis in cancer cells such as HeLa, which show signs of genomic instability without treatment (Fig. S5, a–c). As nuclear blebs, micronuclei, and anaphase bridges are associated with genomic instability and micronuclei are caused by the missegregation of chromosomes during mitosis, these results indicate that inhibition of the synthesis of LCBs compromises the integrity of the nuclear membrane, causing significant dysregulation of the pathways that regulate genome integrity.

Since micronuclei are a product of erroneous chromosome segregation, we monitored this process upon SPT knockdown. To this end, we arrested cells in G2 with the Cdk1 inhibitor RO-3306 and released them into mitosis. Immunofluorescence 45 min after releasing cells from the arrest revealed that while control cells show proper chromosome segregation, targeting SPT leads to abnormal chromosome alignment and microtubule morphology during metaphase (Fig. 9, g and h; and Fig. S5, b and c). Remarkably, our analysis revealed that a significant number of mitotic cells show multipolar mitosis and more than two centrosomes (Fig. 9, g and i). These results indicate that disruption of nuclear integrity may cause genomic instability by indirectly disrupting the regulation of centrosome localization and the duplication cycle affecting their function.

**Figure 10.**
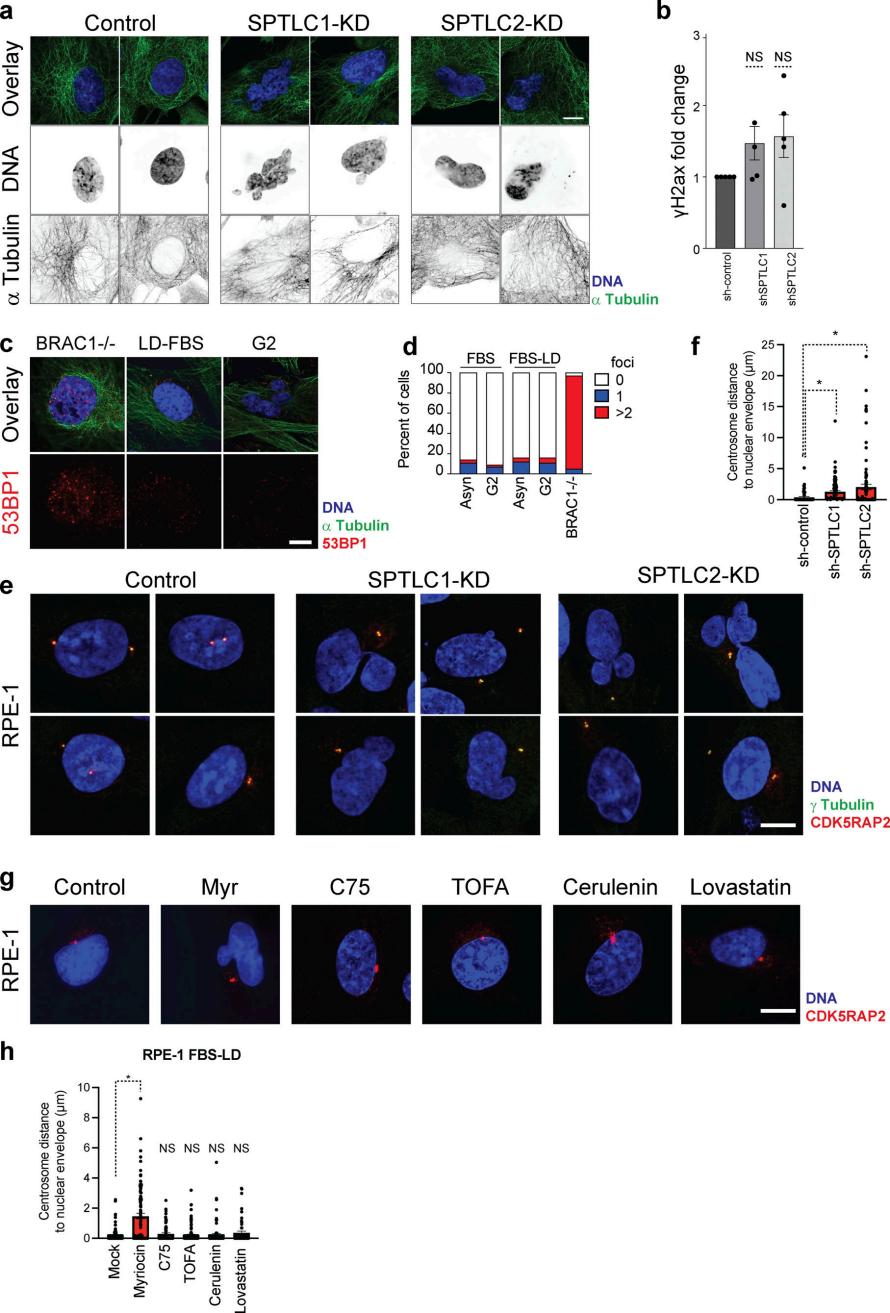
**Centrosome position is affected by lowering the levels of LCBs. (a)** Representative immunofluorescence images of RPE-1 cells upon knockdown of scramble (Control), *SPTLC1*, or *SPTLC2* cultured for 96 h, or treated with 5 µM myriocin or 10 µM fumonisin B_1_ in lipid-depleted medium for 48 h. Green, anti-alpha-tubulin; purple, Hoechst 33342 (DNA); and red, anti-lamin B1. Scale bar, 5 µm. **(b)** Quantification of yH2AX in RPE-1 cells upon knockdown of scramble (Control), *SPTLC1*, or *SPTLC2*. *N* = 5 western blots, NS, P > 5 e-2, one-way ANOVA. **(c)** Representative immunofluorescence images of RPE-1 cells with BRCA1 deletion (left) showing increased 53BP1 foci (red). RPE-1 cells cultured in LD-FBS (middle) or arrested in G2 in LD-FBS with abnormal nuclear morphology (right) do not show 53BP1 foci. Scale bar, 5 µm. **(d)** Quantification of cells shown 53BP1 foci (*n* > 200). **(e)** Representative immunofluorescence images of RPE-1 cells upon knockdown of scramble (Control), *SPTLC1*, or *SPTLC2* cultured for 96 h. Scale bar, 10 µm. **(f)** Quantification of the centrosome distance to the NE in RPE-1 cells. *P < 1 e-4, one-way ANOVA. **(g)** Representative immunofluorescence images of RPE-1 cells treated with 5 µM myriocin, 40 µM C75, 10 µM TOFA, 10 µM cerulenin, or 1 µM lovastatin cells for 24 h. Scale bar, 10 µm. **(h)** Quantification of the centrosome distance to the NE in RPE-1 cells. *P < 1e-4, one-way ANOVA. NS, P > 0.05.

To investigate whether disruption of the cytoskeleton is associated with abnormal nuclear morphology upon lowering the levels of LCBs, we visualized actin filaments and microtubule networks and did not detect significant differences between control cells and cells harboring an affected nucleus (Fig. S4 c and Fig. 10 a). To assess whether chromatin is affected by low levels of LCBs, we evaluated the levels of DNA damage in cells with an abnormal nucleus. The levels of yH2AX or the number of foci of 53BP1 does not significantly increase in cells with abnormal nuclear morphology (Fig. 10, b–d). Instead, we found that the centrosome localization is affected in cells with lowered LCBs. Analysis of the centrosome distance to the nucleus in RPE-1 cells shows that most duplicated centrosomes are juxtaposed to the NE (Fig. 10, e and f). The distance of the centrosome to the nucleus increases upon SPT knockdown, with most duplicated centrosomes being detached and, in some instances, several microns apart from the NE (Fig. 10, e and f). Consistently, the distance of the centrosome to the NE increases upon myriocin treatment but does not change upon inhibition of ACC1, FAS, or HMGCR (Fig. 10, g and h). Together, these results suggest that a possible mechanism by which lowering the levels of LCBs leads to chromosome missegregation is that abnormal nuclear membrane integrity interferes with the proper regulation of the centrosome duplication cycle. This cycle is tightly regulated in time and space, and centrosome mislocalization may lead to abnormal centrosome duplication and maturation necessary to ensure proper chromosome segregation. Further studies will be required to assess centrosome composition upon detachment from the NE during cell division.

## Discussion

In addition to being metabolic precursors of ceramides and complex sphingolipids, LCBs function as signaling molecules regulating cell death and survival. However, specific molecular targets of LCBs have been challenging to identify, and the mechanisms by which they activate a particular signaling pathway remain to be determined as these molecules are embedded in the membrane. Our data support the hypothesis that in addition to functioning as signaling molecules, LCBs play an essential structural role in maintaining the integrity of the nuclear membrane. In yeast, we show that loss of function of sphingosine kinase *LCB4* or ceramide synthase *LAG1*, which have minimal effects on cellular fitness, causes significant accumulation of LCBs (four- to sixfold) in the nucleus. This increase in the levels of LCBs does not affect growth rate, cell viability, or gene expression, indicating the major signaling pathways are not engaged (Giaever et al., 2002). Instead, we find that the nuclear volume increases relative to control cells. While the physiological relevance of an increased nuclear volume is unclear, we previously showed that these increases can suppress nuclear abnormalities caused by increasing the number of chromosomes in the nucleus in aneuploid yeast cells. LCBs also regulate nuclear membrane integrity in human cells. Treating trisomy human fibroblasts that harbor abnormal nuclear morphologies with an inhibitor of ceramide synthase causes a fivefold increase in LCBs, suppresses this phenotype, and improves cellular fitness (Hwang et al., 2019).

We previously showed up to 80% of cellular LCBs are present in the nucleus of yeast cells (Hwang et al., 2019). Here, we present results supporting the hypothesis that LCBs are also enriched in the NE/ER in human cells. Firstly, exogenous fluorescent-labeled LCB molecules get internalized and accumulate in the NE/ER and Golgi apparatus, not in mitochondria, lysosomes, or plasma membrane in human cells. In contrast, ceramides accumulate in the plasma membrane and hardly stain the NE. Secondly, lipidomics shows that most cellular LCBs are in the nucleus in T cells. One hypothesis is that LCBs remain at the site of their synthesis by SPT and KDSR, which localized at the NE/ER. Instead, ceramides, made by adding a second lipid tail to LCBs by ceramide synthase, mobilize out of the ER to serve as substrates for complex sphingolipids. Key biochemical differences between LCBs and ceramides that can influence their localization within the cell include the lipid tail (single versus double) and the charge of the head group (positive versus neutral). Interestingly, yeast cells divide by closed mitosis and have higher levels of LCBs relative to ceramides, while in human cells, LCBs are one tenth the levels compared with ceramide. Since yeast cells do not express lamin proteins, an attractive hypothesis is that higher levels of LCBs are required to maintain the nuclear integrity in yeast during closed mitosis. In support of this, we showed that inhibition of SPT in yeast causes nuclear membrane collapse as cells start to divide their nucleus during early anaphase, and this phenotype strongly correlates with the subsequent loss of viability. SPT is essential in human cells; therefore, we lowered SPT activity by RNA interference or chemical inhibition, and both approaches affected the morphology of the nucleus. These data suggest that LCBs play an essential structural role in the nuclear membrane. We hypothesize that its single-chain composition and positively charged amine group give these molecules specific properties relative to other lipid species to maintain the structure and biophysical properties of the nuclear membrane. Indeed, increases in LCBs affect membrane dynamics in yeast (Hwang et al., 2019).

How cells coordinate membrane expansion with other cell cycle events, such as genome duplication and segregation, is not understood. Surprisingly, genes involved in lipid synthesis are not enriched in cell cycle–regulated genes (Litsios et al., 2024). One possibility is that cells do not upregulate genes involved in lipid metabolism during cell division because most experimental conditions supplement exogenous lipids in the growth medium. Yeast cells grown in rich media or human cells cultured in the presence of serum may exclusively utilize exogenous lipids to divide. Yet, analysis of the cell cycle in yeast grown in minimal media did not reveal genes in lipid synthesis to be cell cycle–regulated, even though metabolomics showed an increase in lipid molecules during the S phase (Campbell et al., 2020). Here, we performed lipidomics of yeast cells grown in minimal media and showed that LCBs are synthesized during the S and G2 phases of the cell cycle. Similarly, human cells arrested in S or G2 phases show increases in the synthesis of LCBs. Remarkably, in human cells, the levels of LCBs precisely doubled together with increases in nucleus volume in late G2, just before the NE breaks down during prophase. Instead, ceramides increase severalfold, sixfold in yeast and threefold in human cells, consistent with previous studies implicating high ceramide levels are required during cytokinesis. Live-cell microscopy following SPT inhibition revealed that abnormal nuclear morphology arises following NE breakdown during the assembly of the new NE in the daughter cells. This observation supports the hypothesis that low levels of LCBs do not cause a cell cycle arrest but are required for proper nuclear membrane integrity during cell division. How LCB synthesis is regulated during the cell cycle remains an important question. One possibility is that the enzymatic activity of SPT, KDSR, or DEGS1 is cell cycle–regulated, as their expression does not significantly change during the cell cycle. In yeast, substrate availability, such as the amino acid serine or palmitate, appears to regulate the levels of LCBs (Cowart and Hannun, 2007; Hwang et al., 2017). Orm1 and Orm2 in yeast and ORMDL1, ORMDL2, and ORMDL3 in humans have emerged as key modulators of SPT activity and constitute potential cell cycle targets to influence sphingolipid synthesis (Davis et al., 2018). Nevertheless, several studies support the hypothesis that general lipid synthesis increases upon entry into the cell cycle, and the protein levels of FAS and ACC increase via posttranscriptional mechanisms to support this (Blank et al., 2017; Campbell et al., 2020). More quantitative studies will be required to establish how specific classes of lipids are regulated during cell division, including measuring the metabolic flux of precursor molecules into de novo synthesis lipids.

Targeting FA synthesis by inhibiting ACC or FAS, inhibiting TOR kinase, which is thought to act upstream of ACC and FAS, or inhibiting cholesterol biosynthesis by statins does not affect the morphology of the nucleus. Quantitative lipidomics shows that the lipid composition of human cells is minimally affected when cells are grown in a lipid-depleted medium, consistent with the hypothesis that robust mechanisms ensure constant levels of lipids in the cell. Indeed, yeast cells grown under several conditions show robust regulation of intracellular lipid levels (Klose et al., 2012). These observations indicate that checkpoints to ensure proper lipid levels are regulated independent of the availability of exogenous lipids. Remarkably, our data show that inhibition of sphingolipid synthesis does not induce cell cycle arrest in yeast or human cells. In yeast, we showed that upon SPT inhibition, cells proceed into the cell cycle and show normal DNA synthesis, bud formation, and regulation of cyclin levels. However, during mitosis, the nuclear membrane is compromised. In human cells, SPT knockdown minimally affects cell proliferation, and RPE-1 cells can double several times, showing abnormal nuclear morphology and, in subsequent cell divisions, signs of chromosomal instability. Despite the lack of sphingolipid synthesis, why cells continue dividing is puzzling. Our studies indicate that the levels of LCBs during mitosis are dependent on at least two factors: the availability of exogenous lipids and the activity of Cdk1, as cells grown in lipid-depleted medium do not increase LCBs upon Cdk1 inhibition coupled with defects in the integrity of the nuclear membrane. An important question remains: how is the balance between the de novo synthesis of lipids and the regulation of lipid uptake from exogenous sources regulated?

Several pathologies associated with genomic instability are studied by targeting different pathways. Inhibition of the spindle assembly checkpoint causes chromosome missegregation and micronuclei (Thompson and Compton, 2011), targeting telomere maintenance causes anaphase bridges and chromothripsis (Umbreit et al., 2020; van Steensel et al., 1998), polo kinase 4 overexpression causes multipolar mitosis (Ganem et al., 2009), and lowering lamin levels causes abnormal nuclear morphology (Hatch et al., 2013). All these pathologies are hallmarks of cancer cells, but what causes these phenotypes in vivo remains unknown. Our study suggests that uncontrolled cell division in an environment with poor nutrient availability, where sphingolipid synthesis is insufficient, could disrupt the nuclear membrane integrity and induce several traits of genomic instability. The mechanisms by which inhibition of sphingolipid synthesis causes genomic instability urge further investigation. Our results indicate that the effects on genome maintenance are an indirect consequence of nuclear membrane disruption. The immediate result of SPT inhibition is abnormal nuclear morphology, and signs of genomic instability appear only after two or three population doublings. One possibility is that abnormal lipid composition of the nucleus disrupts the regulation of the centrosome duplication cycle. Centrosomes, which consist of hundreds of proteins (O’Neill et al., 2022), are tightly regulated and closely associated with the NE during mitosis (Bolhy et al., 2011; Nigg, 2007). Abnormal centrosome function may account for the dysregulation of microtubule polymerization and their overduplication during multipolar mitosis. Lastly, abnormal nuclear morphology is a hallmark of aging, premature aging, and aneuploidy, including trisomy 21, which causes Down’s syndrome. At least, in the case of aneuploid cells, sphingolipid levels are affected, which raises the possibility that this phenotype may drive genomic instability in these cells.

## Materials and methods

### Yeast strains and growth conditions

All stains are derivatives of W303 (E187, *MATa/MATα*, *ade2-1*, *leu2-3*, *ura3*, *trp1-1*, *his3-11,15*, *can1-100*, *GAL*, *psi*^*+*^). Deletions were introduced in WT cells by transformation using a PCR method that replaces the desired gene by a selectable marker (yeast Pringle primer method): *lcb4∆* delete (E460, *LCB4::CaURA3*), *lag1∆* (E413, *LAG1::CaURA3*), *tsc3∆* (E1166, *TSC3::CaURA3*). *HEH1* was tagged with GFP at the C terminus (E1330, *HEH1::HEH1-GFP-URA3*). One liter of minimal medium consists of 1.7 g of yeast nitrogen base without amino acids or ammonium sulfate (#233520; BD), 4.8 g of ammonium sulfate (#A4418; Sigma-Aldrich), and 2 g of amino acid mix, which consist of equal amounts of Ala #A7469, Arg #A8094, Asn #A0884, Asp #A4534, Cys #C7352, Gln #G3126, Glu #G1251, Gly (G7403), His #H8125, Iso #I2752, Leu #61819, Lys #L5501, Met #M9625, Phe #P2126, Pro #P5607, Ser #S4311, Thr #T8625, Trp #T0254, Tyr #T3754, Val #V0500, adenine #A9126, and uracil #U0750 purchased from Sigma-Aldrich.

### Live-cell microscopy of yeast cells

Cells grown at 25°C in synthetic media were collected during early exponential phase (OD_600_ between 0.6 and 1.0) by centrifugation. After washing cells with 1X phosphate-buffered saline (PBS), they were put on slide glass and covered with glass and images were immediately taken using Nikon Eclipse E400 compound microscope. 100× 1.45 oil magnification lens, Andor Clara-E Camera (DR-328G-C02-SIL), and NIS-Elements imaging software were used.

### Quantification of nuclear volume from microscopy

Images were analyzed using ImageJ software (https://imagej.nih.gov). Using the freehand tool, the surface area of 200 nondividing nuclei was measured in pixels. The mean value was calculated by fitting the histogram to a normal distribution using GraphPad Prism software.

Electron microscopy of WT cells and cells harboring *tsc3∆* was outsourced to the Harvard Medical School EM Facility. Leica EM ICE High Pressure Freezer was used.

### Quantification of abnormal nuclear morphology in yeast

To quantify the number of abnormal nuclei, 200 nondividing cells expressing Heh1-GFP were analyzed by live-cell microscopy. Normal nuclei that are always observed in WT cells show mostly circular, smooth, and continuous GFP signal. Any nucleus that showed deformed shape, broken nuclei, aggregated GFP signal, invaginations, membrane expansions, or discontinuous GFP signal was considered abnormal.

### Volume quantification of purified nuclei using Coulter counter

Yeast cells grown at 25°C in synthetic media were collected during early exponential phase (OD_600_ between 0.6 and 1.0). 10^8^ cells were harvested and washed twice with water. Cells were lysed in 1 ml of lysis buffer (1.1 M sorbitol, 20 mM KCl, 0.5 mM MgCl_2_, pH 7.4) with 50 µg/ml of zymolyase (#Z1000; USBiological) and 10 mM DTT for 1 h at 30°C. Spheroplasts were spun down at 370 rcf for 5 min, resuspended in hypotonic solution (8% PVP, 10 mM Tris-HCl, pH 6.4, 0.5 mM MgCl_2_), and incubated for 10 min at room temperature. Nuclei were spun down at 370 rcf for 5 min and resuspended in PBS right before measuring their volume in a Coulter counter (*n* = 30,000). To quantify cell volume, yeast cells grown at 25°C in synthetic media were collected during early exponential phase (OD_600_ between 0.6 and 1.0) and sonicated at low power for 3 s before measuring cell size in the Coulter counter (*n* = 10,000).

### Treatment of yeast cells with drugs

Yeast cells were grown at 25°C in synthetic media, and drugs were added at an OD_600_ of 0.4 and incubated for indicated times before imaging or harvesting for lipid analysis. Myriocin #63150 and cerulenin #10005647 were purchased from Cayman Chemical. Noteworthily, 2.5 µM myriocin was used when the cell density was close to OD_600_ 0.2 including the cell cycle analysis after release from the pheromone arrest.

### Quantification of yeast viability

Cell numbers were quantified using the Coulter counter. Cells were diluted severalfold to the plate between 100 and 200 μl of volume to yield 200 colonies in YEPD agar plates. After 2 days, images of the plates were acquired, and colonies were counted using the ImageJ counter tool.

### Mass spectrometry of sphingolipids

Lipidomics of yeast and human cells was outsourced to the Lipidomics Shares Resource at the Medical University of South Carolina (MUSC). Cells were treated with 5% trichloroacetic acid (TCA, #T9159; Sigma-Aldrich) for 10 min on ice and washed with water three times before shipping in dry ice. Three independent cultures were analyzed for each strain, and relative levels of all lipids were normalized to total cell numbers, which were determined using a Beckman Coulter counter. Protocols for lipid extraction and analysis are described in Bielawski et al. (2009). In short, 1 × 10^6^ cells were extracted in 2.0 ml of isopropanol:water:ethyl acetate (30:10:60) (by vol). Samples were sonicated for 30 s, vortexed, and centrifuged for 5 min at 3,000 × *g*. The organic upper phase was evaporated under nitrogen to dryness. Lipids were reconstituted in 150 µl of 1 mM of ammonium formate in methanol containing 0.2% formic acid and injected into an HPLC system followed by mass spectrometry analysis.

### Gene expression quantification

Cells were grown overnight at 25°C in minimal medium. Batch cultures were diluted to OD_600_ = 0.2 into minimal medium the next day, and when they reached an OD_600_ = 0.6, cells were treated with myriocin and harvested at indicated time points. The RNeasy kit from Qiagen (#74104) was used to purify the RNA, and a NanoDrop (Thermo Fisher Scientific) was used to measure concentration. Samples were shipped for on dried ice for transcriptome sequencing to BGI Americas (https://www.bgi.com/). All RNA-seq reads were mapped to the yeast genome and normalized to the total number of reads per experiment (Transcripts Per Kilobase Million [T.P.M.] reads). To calculate the fold changes among all cell lines, the average T.P.M. per gene was calculated for all the control samples and the average T.P.M. was used as a reference genome. Log_2_ (T.P.M. per gene/T.P.M. of reference) was obtained for each sample. We included FC for genes that were detected in all samples and a cutoff of 1 T.P.M. or greater in our analysis. Hierarchical clustering was performed using the program WCluster. WCluster takes both a data table and a weight table to allow individual measurements to be differentially considered by the clustering algorithm. Gene expression data were clustered by a Pearson correlation metric with equal weighting given to all data. The PRISM software was used to calculate frequency distributions and calculate Pearson’s r correlation coefficients.

### Cell cycle analysis in yeast

Cells were arrested in G1 in synthetic media with 5 µg/ml alpha factor (#Y1001; Zymo Research) for 3.5 h. 2 h into the arrest, 2.5 µg/ml alpha factor was readded. Cells were washed with 10 volumes of synthetic media and released into medium lacking pheromone. An equal number of cells were harvested at each time point, and DNA content was analyzed by Guava easyCyte (Millipore), percentage of budded cells were analyzed using DIC microscopy, and Clb2 levels were analyzed by western blots. Samples for lipidomics analysis were immediately treated with TCA and flash-frozen in liquid nitrogen, and kept at −80°C before shipping them to MUSC lipidomics facility.

### Western blot in yeast

Western blot analysis cells were collected from the release of alpha factor arrest. 10 ml of culture was harvested and lysed in lysis buffer (50 mM Tris-HCl, pH 7.5, 150 mM NaCl, 2 mM DTT, 2 mM EDTA plus protease inhibitors, #P8340; Sigma-Aldrich) using acid-washed glass beads. Lysates were diluted with 3 X sample loading buffer (50 mM Tris-HCl, pH 6.8, 2% SDS, 100 mM B-mercaptoethanol, 0.2% bromophenol blue) and heated at 95°C for 10 min. 50 µg of lysate was loaded into the 15% Tris-HCl protein gels and transferred to a PVDF membrane (IPVH09120; Millipore). Western blots were quantified using ImageJ. For Clb2, we used sc-9071 (Santa Cruz), and for PSTAIR, #P7962 (Sigma-Aldrich). Goat anti-rabbit antibody (Bio-Rad) #1706515 and goat anti-mouse antibody (Bio-Rad) #1721011 were used as secondary antibodies.

### FACS of yeast cells

After fixing yeast cells with 70% ethanol overnight at 4°C, the cells were washed three times with 1× PBS and once with 50 mM sodium citrate. The fixed cells were then sonicated for 3 s at setting 1 using a BRANSON Digital Sonifier 250. Next, they were incubated with 125 µg/ml RNase A in 50 mM sodium citrate for 1 h at 50°C, followed by the addition of 125 µg/ml Proteinase K and an additional 1-h incubation at 50°C. For staining, cells were mixed with 2 µM SYTOX Green (#S7020; Invitrogen) in 50 mM sodium citrate and incubated for 30 min at room temperature. Prior to analysis on a BD FACSCelesta, the cells were sonicated again for 3 s at setting 1.

### FACS of human cells

After fixing trypsinized cells with 70% ethanol overnight at 4°C, the cells were washed once with 1× PBS and resuspended in 0.5% Tween-20/IFA buffer (10 mM HEPES-KOH, pH 7.4, 150 mM NaCl, 4% FBS, 0.1% sodium azide). RNase A was added to a final concentration of 5 µg/ml, and the cells were incubated for 30 min at 37°C. For staining, cells were mixed with 10 µg/ml propidium iodide (PI, #P1304MP; Invitrogen) in 50 mM sodium citrate and incubated for 30 min at room temperature. Cells were then analyzed using a BD FACSCelesta.

### BrdU staining for FACS analysis

After fixing trypsinized cells with 70% ethanol overnight at 4°C, the cells were washed once with 1× PBS, resuspended in 0.5% Triton X-100/2 M HCl, and incubated for 30 min at room temperature. The cells were then washed with 25 mM borax (pH 8.5), resuspended in 1% BSA and 0.05% Tween-20 in 1× PBS, incubated with BrdU antibody conjugated with FITC (#11-5071-42; Invitrogen), and incubated for 1 h at room temperature. Afterward, the cells were washed twice with 1% BSA/PBS and resuspended in 10 µg/ml PI in 1% BSA/PBS. Cells were then analyzed using a BD FACSCelesta.

### Visualization of fluorescent-labeled sphingolipids

HeLa cells were grown in Dulbecco’s modified Eagle’s medium (DMEM, #11995065; Gibco) supplemented with 10% FBS (FBS, #F2442; Sigma-Aldrich). Fluorescent probes were purchased from Avanti Polar Lipids: NBD-Sphinganine (DHS) #81026, NBD-Sph #810205, NBD-18:0-ceramide #810210, NBD-S1P #810207, C6-NBD-ceramide #810209. All probes were added at 1 µM concentration and incubated for 20 min before acquiring live-cell confocal images using a Nikon A1 Eclipse Ti2 inverted microscope equipped with a confocal laser detector Nikon A1R HD25. 100× 1.45 and 60× 1.42 magnification lenses and NIS-elements software were used to acquired and process the images. Selected crossed sections that capture the largest area of the NE are shown in the figures. To visualize the different organelles in HeLa, cells were transfected with the following plasmids obtained from Addgene: LAMP1-mCherry #45147, mCherry-mito-7 #55102, mCherry-Golgi-7 #55052, mCherry-Farnesyl-5 #55045, mCherry-ER-3 #55041.

### T cell isolation

Blood from normal donors was obtained through UMass Leukocyte Core Facility. Our core facility provides leukoreduction filters from whole units of blood obtained from normal blood donors. We then extracted the leukocytes from the leukoreduction filters for the isolation of peripheral blood mononuclear cells (PBMCs). To purify PBMCs, we added 15 ml of Lymphoprep (#07851; StemCell Technologies) to the SepMate tube (#85450; StemCell Technologies) by carefully pipetting it through the central hole of the SepMate insert. Then, blood was diluted with an equal volume of 2% FBS/PBS and mixed gently. The diluted blood was layered over the top of Lymphoprep and centrifuged at 1,200 × *g* for 10 min at RT, and the top supernatant was carefully removed. The cell layer (5–10 ml) was transferred into a new tube, and 30 ml of 2% FBS/PBS was added and centrifuged at 300 × *g* for 8 min at RT, and the supernatant was removed. Cells were resuspended with 5 ml of 2% FBS/PBS, and then, 45 ml of 1 X RBC lysis buffer (0.15 M ammonium chloride, 10 mM potassium bicarbonate, 0.1 mM EDTA) was added, incubated for 5 min on the shaker (85 rpm), and centrifuged at 300 × *g* for 8 min at RT. The supernatant was removed, and 30 ml of 2% FBS/PBS was added and centrifuged at 300 × *g* for 8 min at RT, and the supernatant was removed. A wash was repeated two times. 30 ml of 2% FBS/PBS was added and centrifuged at 120 × *g* for 10 min at RT, and cells were resuspended in 10 ml of 2% FBS/PBS. PBMCs were ready to isolate CD4^+^ T cells.

### CD4^+^ T cell isolation

We used EasySep Human CD4^+^ T Cell Enrichment Kit (19051; StemCell Technologies). Cells were diluted to make around 5 × 10^7^ cells/ml. 5 × 10^7^ cells were added to 1-ml new e-tube. 50 μl of enrichment cocktail was added and mixed well, and incubated for 10 min at RT. 100 μl of magnetic beads was added and mixed well, and incubated for 5 min at RT. e-Tube was put in the magnetic rack and allowed to wait for 5 min at RT. The supernatant (containing CD4^+^ T cells) was transferred into the new tube and centrifuged at 300 × *g* for 10 min at RT. The pellet is CD4^+^ T cells. For lipidomics, cells were washed with PBS three times to remove FBS.

### Nucleus isolation of T cells

∼1 × 10^7^ cells were resuspended in 2 ml of hypotonic solution (10 mM HEPES (pH 7.9), 10 mM KCl, 1.5 mM MgCl_2_), gently mixed by slowly pipetting 15 times, incubated for 5 min at RT, and centrifuged at 2,000 rpm for 5 min at 4°C. The supernatant was removed, and the pellet was carefully resuspended with 3 ml of S1 solution (0.25 M sucrose, 10 mM MgCl_2_). The resuspended pellet was layered over 4 ml of S2 solution (0.25 M sucrose, 0.5 mM MgCl_2_) in 15-ml conical-bottom centrifuge tube and centrifuged at 604 rcf for 5 min at 4°C. All supernatants were removed, and the pellet was resuspended with 3 ml of 0.5 mM MgCl_2_/PBS and centrifuged at 604 rcf for 5 min at 4°C. Nuclei are in the pellet.

### Culture of RPE-1 cell lines

RPE-1 cells were grown in DMEM (Gibco) supplemented with 10% FBS (Sigma-Aldrich). For lipid-depleted media, we used 10% charcoal-filtered FBS (FB-50; Omega Scientific). For the cell cycle arrest, drugs were added to 3 × 10^5^ and incubated with 2 µM palbociclib (#PD 0332991; Cayman), 2 µM thymidine (#T9250; Sigma-Aldrich), or 10 μM RO3306 (#SML0569; Sigma-Aldrich). Torin 1 (Cayman) #10997, TOFA (Cayman) #10005263, cerulenin (Cayman) #10005647, C75 (Cayman) #9000783, and lovastatin (Cayman) #10010338 were used to inhibit lipid synthesis at the indicated concentrations. To knock down SPTLC1 and SPTLC2, we used methods in Hwang et al. (2019). For SPTLC1, we used pLKO.1:TRCN0000035010 Open BioSystems, for SPTLC2, pLKO.1:TRCN0000034973 Open BioSystems, and for control scramble, pLKO.1-scramble Addgene #1864, RPE-1 (hTERT-RPE-1, ATCC #CRL-4000 TM) and HeLa (#CCL-2TM; ATCC).

### Immunofluorescence microscopy

Cells were fixed with methanol 10 min and permeabilized with 0.1% of Triton X-100 in 1X PBS for 8 min at room temperature. After blocking with 5% BSA in 1X PBS for 1 h at room temperature, cells were incubated with 0.2 μg/ml of primary antibodies in 1X PBS for 2 h at room temperature. Primary antibodies and nuclei were visualized by donkey anti-mouse IgG H&L Alexa Fluor 488 (#ab150105; Abcam), donkey anti-rabbit IgG H&L Alexa Fluor 568 (#ab175470; Abcam), and Hoechst 33342 (#H3579; Invitrogen). For F-actin staining, Phalloidin-iFluor 488 (ab176753; Abcam) was used. Fluorescence images were acquired with a Nikon A1 Eclipse Ti2 microscope. A representative z-slice from the image stack was chosen for figures. For lamin B1, we used Abcam #ab16048, for lamin A/C, we used Abcam #ab108595, for CDK5RAP2, we used Millipore #06-1,398, for alpha-tubulin, we used Santa Cruz #sc-8035, and for gamma-tubulin, we used Abcam #ab27074. Confocal lens, 100× and 60×, were used. 37°C was used for live-cell, and room temperature was used for fixed samples. Cargille Immersion Oil Type LDF # 16241 was used. Live-cell imaging was done in 10%FBS/DMEM or 10% LD-FBS/DMEM. Nikon software NIS-Elements Denoise.ai was used to process images.

### Nanolive imaging

Images were acquired using the Nanolive 3D explorer (https://www.nanolive.com/). Cells were cultured at 37°C. RPE-1 cells were grown in lipid-depleted media, and 10% charcoal-filtered FBS (FB-50; Omega Scientific) was used.

### Western blot assay

For human samples, cells were lysed in lysis buffer (25 mM Tris-HCl, pH 7.5, 150 mM NaCl, 1% Triton X-100, 0.1% SDS, and 0.5% deoxycholate with protease inhibitors). The lysate was separated on SDS polyacrylamide gels, and then, proteins were transferred onto a PVDF membrane (#IPVH.00010; Millipore) and analyzed with the indicated antibodies against: calnexin (Santa Cruz) #sc-23954, NDUFS3 (Abcam) #ab110246, PSPH (Abcam) #ab96414, lamin B1 (Abcam) #ab16048, histone H3 (Millipore) #07-690, SPTLC1 (Abcam) #ab176706, SPTLC2 (Abcam) #ab176706, and 53BP1 (Cell Signaling) #4937. Immunoreactive signals were detected by the SuperSignal West Pico PLUS (#34580; Thermo Fisher Scientific). Goat anti-rabbit antibody (Bio-Rad) #1706515 and goat anti-mouse antibody (Bio-Rad) #1721011 were used as secondary antibodies.

### RNA-seq of human cells

Cells were grown for 48 h, and 10^6^ cells were harvested between 50% and 70% confluency. The RNeasy Kit from Qiagen (#74104) was used to purify the RNA, and a NanoDrop was used to measure concentration. Samples were shipped for on dried ice for transcriptome sequencing to BGI Americas (https://www.bgi.com/). Paired-end reads were aligned to human genome assembly T2T-CHM13v2.0 (Homo_sapiens-GCA_009914755.4-softmasked.fa) (Aganezov et al., 2022), with star_2.5.3a 1, and annotated with Ensembl annotation released on 2022_07 (Homo_sapiens-GCA_009914755.4-2022_07-genes.gtf) (Martin et al., 2023). Aligned exon fragments with mapping quality >20 were counted toward gene expression with featureCounts_1.5.2 3. Expression normalization was performed using the T.P.M. method (Dobin et al., 2013; Harrow et al., 2012; Liao et al., 2014). The same RNA-seq analysis workflow was used as described above for yeast.

### Online supplemental material

Fig. S1 shows synthesis of LCBs determine nuclear shape and volume in yeast. Fig. S2 shows LCBs are integral components of the nuclear membrane in human cells. Fig. S3 shows inhibition of SPT disrupts nuclear morphology in human cells. Fig. S4 shows LCBs are synthesized during S and G2 phases in human cells. Fig. S5 shows lack of LCB increases genomic instability in cancer cells. Table S1 shows lipidomics of myriocin-treated yeast cells. Table S2 shows RNA-seq of yeast cells treated with myriocin for 0, 60, or 180 min. Table S3 shows lipidomics of yeast cells after alpha factor release with no myriocin or 2.5 µM myriocin. Table S4 shows lipidomics of RPE-1 cells. Table S5 shows RNA-seq of RPE-1 cells in FBS or LD-FBS. Video 1 shows live time-lapse fluorescence microscopy of RPE-1 expressing GFP-histone H2B in LD-FBS without any drugs. Video 2 shows live time-lapse fluorescence microscopy of RPE-1 expressing GFP-histone H2B in LD-FBS with 5 µM myriocin. Video 3 shows nanolive live-cell microscopy of RPE-1 cells in LD-FBS. Video 4 shows nanolive live-cell microscopy of RPE-1 cells in LD-FBS with 5 µM myriocin.

## Supplementary Material

Table S1 shows lipidomics of myriocin-treated yeast cells.

Table S2 shows RNA-seq of yeast cells treated with myriocin for 0, 60, or 180 min.

Table S3 shows lipidomics of yeast cells after alpha factor release with no myriocin or 2.5 µM myriocin.

Table S4 shows lipidomics of RPE-1 cells.

Table S5 shows RNA-seq of RPE-1 cells in FBS or LD-FBS.

SourceData F4 is the source file for Fig. 4.

SourceData F5 is the source file for Fig. 5.

SourceData FS3 is the source file for Fig. S3.

SourceData FS5 is the source file for Fig. S5.

## Data Availability

Data are provided as in the supplemental tables: Table S1 shows lipidomics of myriocin-treated yeast cells. Table S2 shows RNA-seq of yeast cells treated with myriocin for 0, 60, or 180 min. Table S3 shows lipidomics of yeast cells after alpha factor release with no myriocin or 2.5 µM myriocin. Table S4 shows lipidomics of RPE-1 cells. Table S5 shows RNA-seq of RPE-1 cells in FBS or LD-FBS. Reagents were obtained from commercial sources as indicated. This study did not generate new unique reagents. Further information and requests for resources and reagents should be directed to and will be fulfilled by the Lead Contact, Eduardo Torres (eduardo.torres@umassmed.edu).
