# Supplementary figures and images for: Sphingolipid synthesis maintains nuclear membrane integrity and genome stability during cell division
Source: J Cell Biol. 2025 Jul 3;224(8):e202407209. doi: 10.1083/jcb.202407209 (PMC12237252; doi:10.1083/jcb.202407209)

Fig 4b

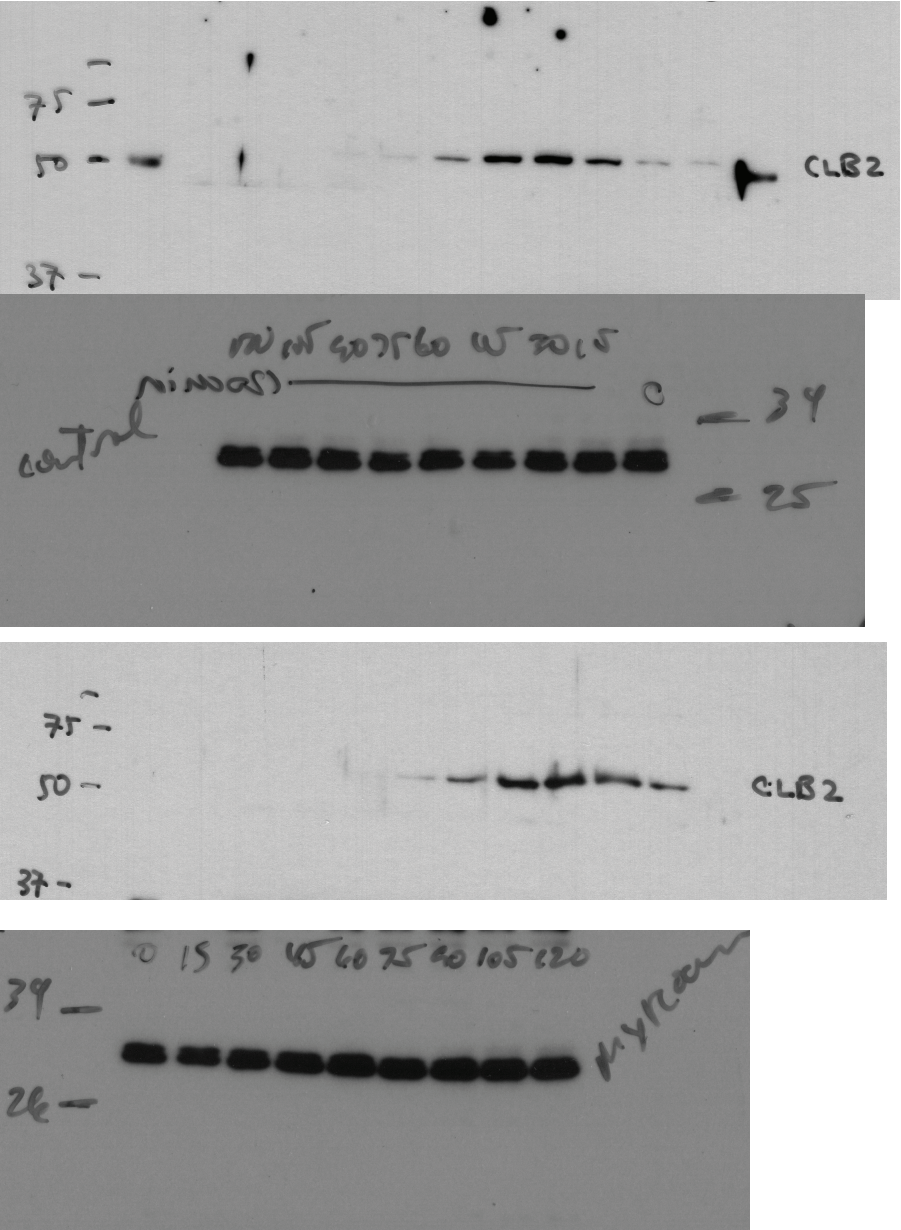

Fig 4i

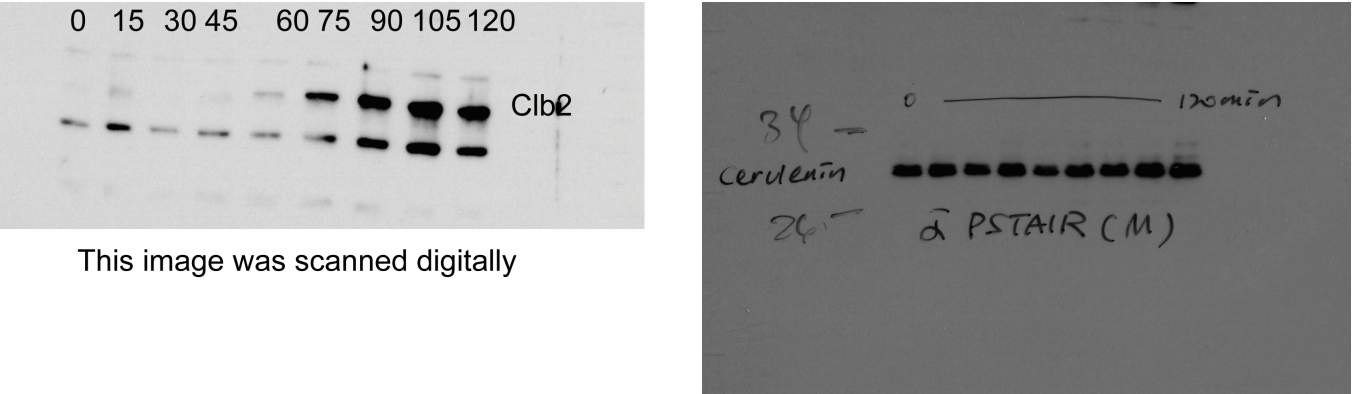

This image was scanned digitally

Supplement: SourceData F4 — is the source file for Fig. 4. [file jcb_202407209_sourcedataf4.pdf]

Fig. 5i

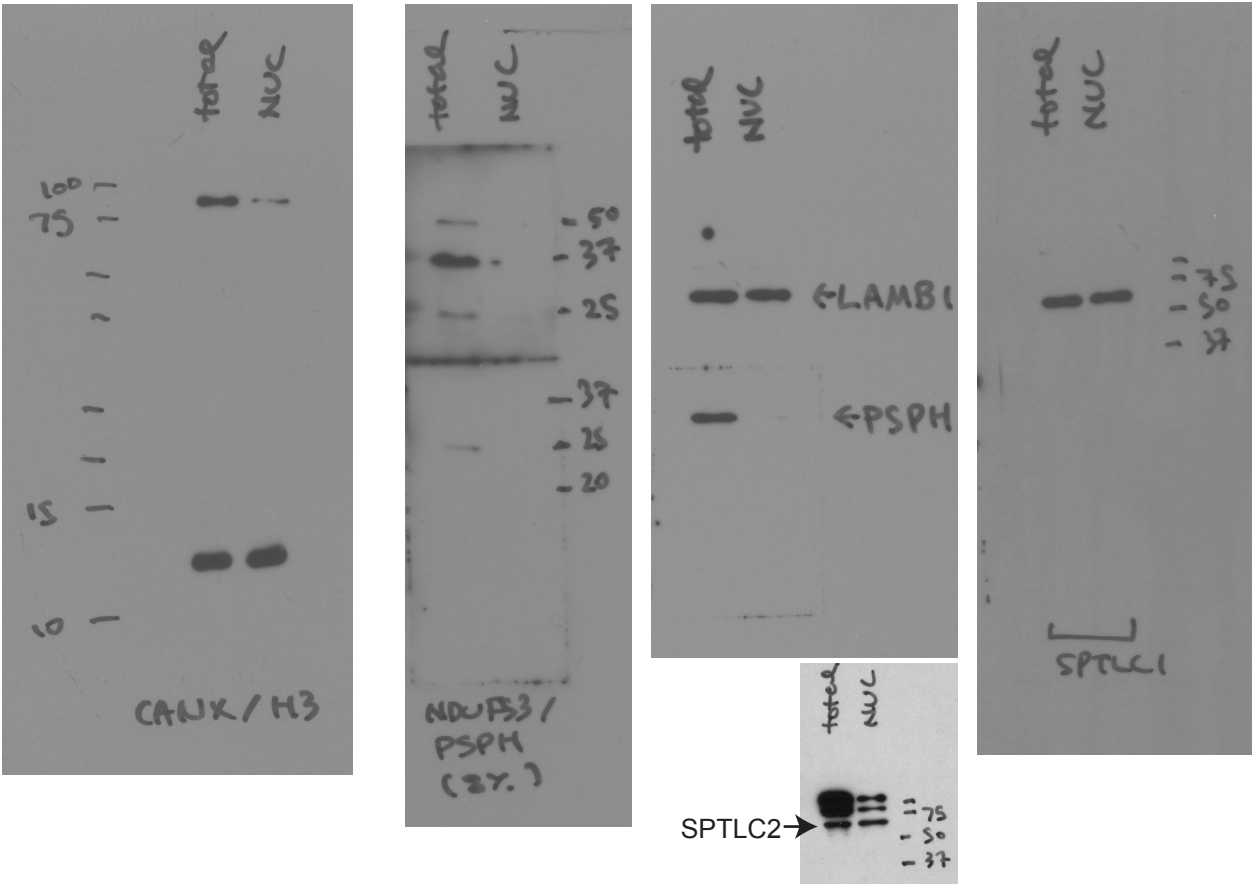

Supplement: SourceData F5 — is the source file for Fig. 5. [file jcb_202407209_sourcedataf5.pdf]

Fig. S3a

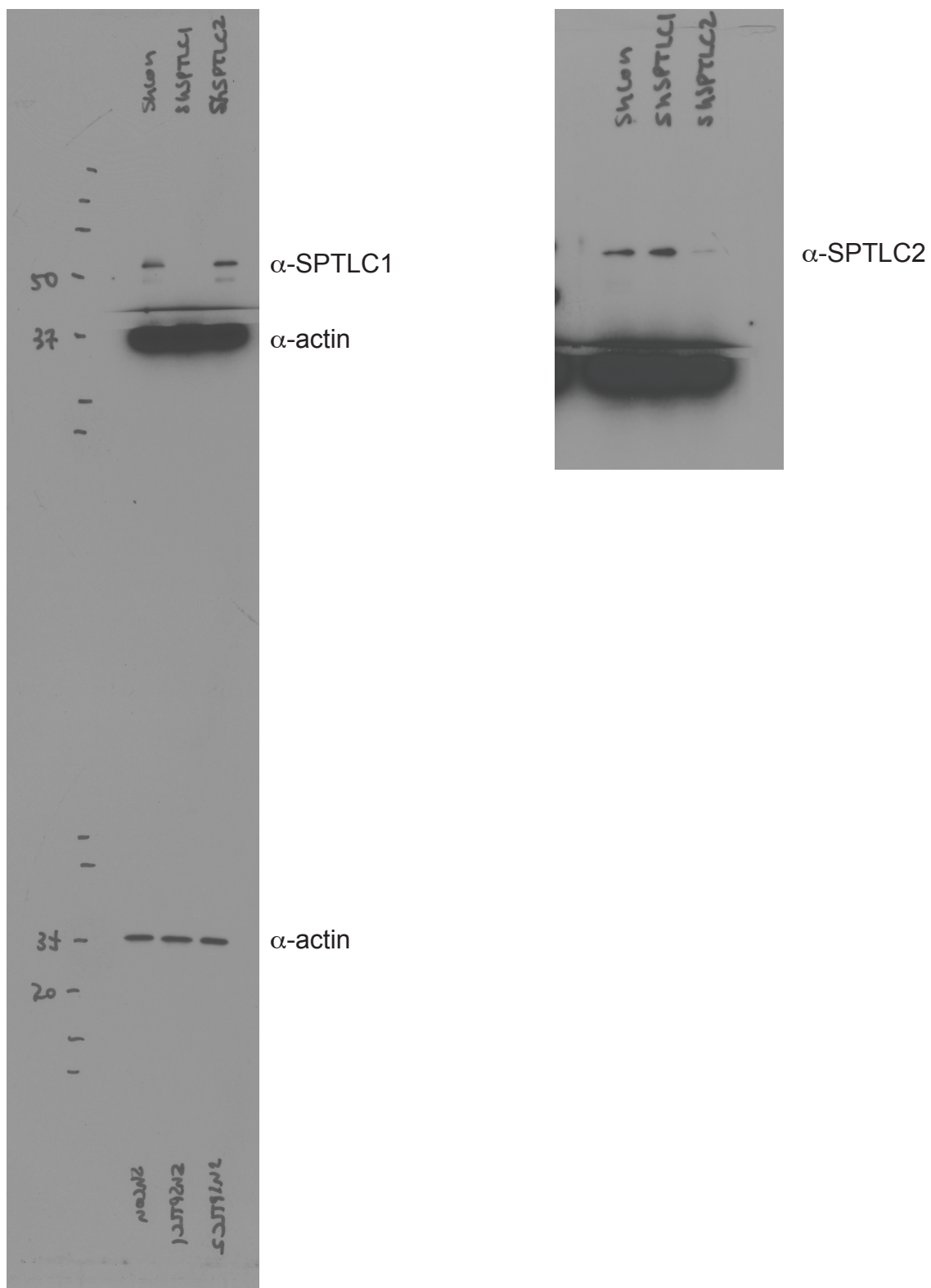

Supplement: SourceData FS3 — is the source file for Fig. S3. [file jcb_202407209_sourcedatafs3.pdf]

## HeLa

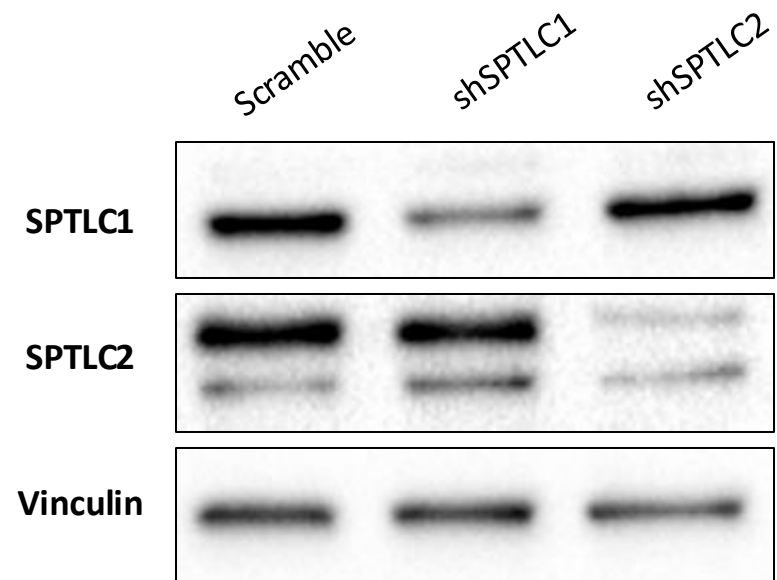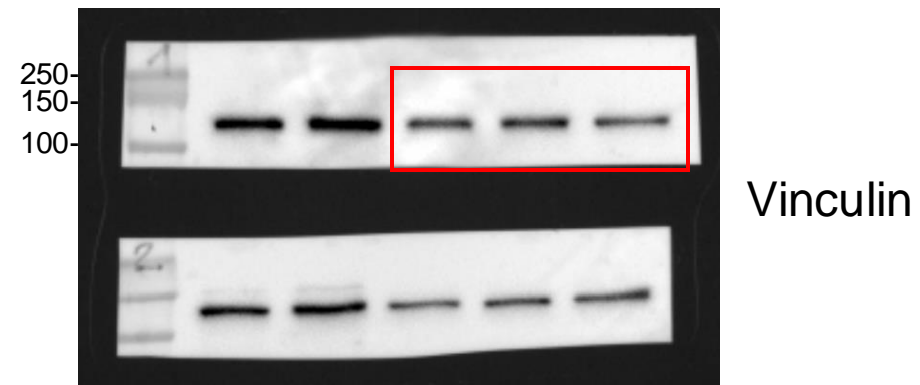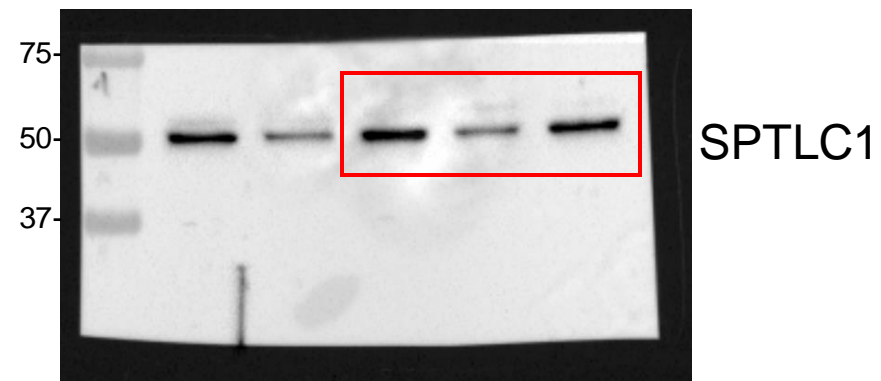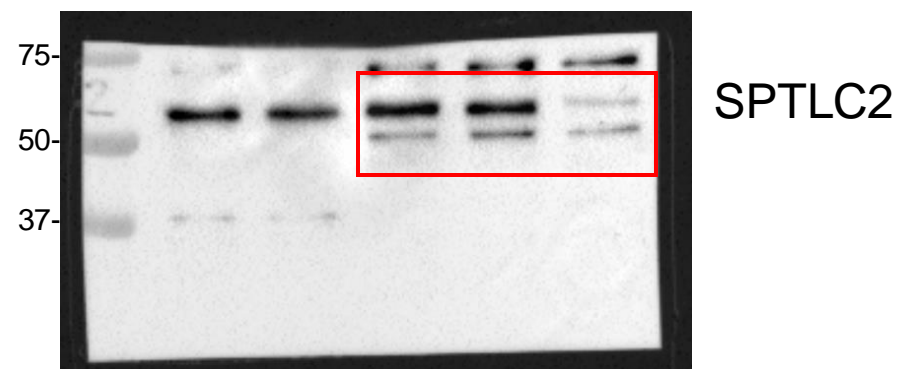

Supplement: SourceData FS5 — is the source file for Fig. S5. [file jcb_202407209_sourcedatafs5.pdf]
